# Unraveling climate change-induced compound low-solar-low-wind extremes in China

**DOI:** 10.1093/nsr/nwae424

**Published:** 2024-11-25

**Authors:** Licheng Wang, Yawen Liu, Lei Zhao, Xi Lu, Liangdian Huang, Yana Jin, Steven J Davis, Amir Aghakouchak, Xin Huang, Tong Zhu, Yue Qin

**Affiliations:** College of Environmental Sciences and Engineering, Peking University, Beijing 100871, China; Institute of Carbon Neutrality, Peking University, Beijing 100871, China; School of Atmospheric Sciences, Nanjing University, Nanjing 210023, China; Department of Civil and Environmental Engineering, University of Illinois at Urbana-Champaign, Urbana, IL 61801, USA; National Center for Supercomputing Applications, University of Illinois at Urbana-Champaign, Urbana, IL 61801, USA; School of Environment, State Key Joint Laboratory of Environment Simulation and Pollution Control, Tsinghua University, Beijing 100084, China; College of Environmental Sciences and Engineering, Peking University, Beijing 100871, China; Institute of Carbon Neutrality, Peking University, Beijing 100871, China; College of Environmental Sciences and Engineering, Peking University, Beijing 100871, China; Institute of Carbon Neutrality, Peking University, Beijing 100871, China; Department of Earth System Science, University of California, Irvine, Irvine, CA 92697, USA; Department of Earth System Science, University of California, Irvine, Irvine, CA 92697, USA; Department of Civil and Environmental Engineering, University of California, Irvine, Irvine, CA 92697, USA; School of Atmospheric Sciences, Nanjing University, Nanjing 210023, China; College of Environmental Sciences and Engineering, Peking University, Beijing 100871, China; Institute of Carbon Neutrality, Peking University, Beijing 100871, China; College of Environmental Sciences and Engineering, Peking University, Beijing 100871, China; Institute of Carbon Neutrality, Peking University, Beijing 100871, China

**Keywords:** wind energy, solar energy, renewable energy, climate change, compound energy droughts

## Abstract

China's pursuit of carbon neutrality targets hinges on a profound shift towards low-carbon energy, primarily reliant on intermittent and variable, yet crucial, solar and wind power sources. In particular, low-solar-low-wind (LSLW) compound extremes present a critical yet largely ignored threat to the reliability of renewable electricity generation. While existing studies have largely evaluated the impacts of average climate-induced changes in renewable energy resources, comprehensive analyses of the compound extremes and, particularly, the underpinning dynamic mechanisms remain scarce. Here we show the dynamic evolution of compound LSLW extremes and their underlying mechanisms across China via coupling multi-model simulations with diagnostic analysis. Our results unveil a strong topographic dependence in the frequency of compound LSLW extremes, with a national average frequency of 16.4 (10th–90th percentile interval ranges from 5.3 to 32.6) days/yr, when renewable energy resources in eastern China are particularly compromised (∼80% lower than that under an average climate). We reveal a striking increase in the frequency of LSLW extremes, ranging from 12.4% under SSP126 to 60.2% under SSP370, primarily driven by both renewable energy resource declines and increasingly heavily-tailed distributions, resulting from weakened meridional temperature (pressure) gradient, increased frequency of extremely dense cloud cover and additional distinctive influence of increased aerosols under SSP370. Our study underscores the urgency of preparing for significantly heightened occurrences of LSLW events in a warmer future, emphasizing that such climate-induced compound LSLW extreme changes are not simply by chance, but rather projectable, thereby underscoring the need for proactive adaptation strategies. Such insights are crucial for countries navigating a similar transition towards renewable energy.

## Teaser

This work represents the spatiotemporal patterns and the underlying drivers of compound low-solar-low-wind extremes over time across China. These insights are pivotal in facilitating low-carbon energy transition, thereby supporting China’s pursuit of carbon neutrality.

## INTRODUCTION

Amidst the increasing global consensus to reduce greenhouse gas emissions in response to anthropogenic climate change, China made an ambitious pledge to go carbon neutral. Such an ambitious pledge requires a progressive transition from a coal-dominated energy system (e.g. 57% of China's primary energy consumption) to one dominated by non-fossil energy (e.g. 80% of China's primary energy consumption) [1,2], supported in particular by solar photovoltaic (PV) (∼37.5%) and wind (∼36%) power [3]. Prior studies have consistently highlighted substantial renewable energy resource potential in China. In recent years, there has been significant growth in both installed capacity and electricity generation for solar and wind power due to an accelerated clean energy transition [4–7].

Renewable energy, unlike traditional fossil fuels, is highly sensitive to meteorological conditions under ever-changing weather and climatic systems. Both wind and solar power exhibit sensitivity to local meteorological factors including wind speed, ambient temperature and solar radiation [8–14]. Because of the fast-evolving weather, wind and solar power generation are often criticized for being variable and intermittent. In particular, extreme meteorological conditions, such as storms and thick clouds can largely threaten solar power generation, while thunderstorms and typhoons will significantly imperil wind power availability [15–17]. The failure of isolated solar or wind power resources can pose substantial challenges to reliable electricity availability, a concern that can be further exacerbated by meteorology-induced concurrent low-solar and low-wind (LSLW) resource availability. We thereby introduce the concept of compound LSLW extremes hereafter (also referred to as *compound solar and wind droughts* in some studies [18,19]). Moreover, as climate change can affect renewable energy resources via reshaping the distribution of renewables-relevant meteorological variables, it could therein affect both the frequency and intensity of compound events relevant to renewable generation. Consequently, this could heighten the threat to China's future renewable energy availability across its seven regional power grids (Fig. S1) and potentially impede its progress towards carbon neutrality.

Considerable effort has been made to evaluate solar or wind resources and their variability at both the regional and global scales for the historical period, under average or in a few cases extreme climate change [12,20–22]. Earlier observational data sets show that solar radiation has experienced periods from dimming to brightening [23–25], while surface wind speed has exhibited a decreasing trend in recent decades across China [26–29]. As the climate warms in the future, projected surface wind speed will decrease markedly under most shared socioeconomic pathway (SSP) scenarios in China [27], and nationally averaged PV power potential decreases significantly under the SSP585 scenario but increases under the SSP126 scenario [21]. Recent studies also highlight that wind and solar energy resources individually will increase with decreasing variability across China upon achieving the carbon neutrality target [5,30]. Unlike the more widely evaluated individual renewable energy resource, little is known regarding the spatiotemporal dynamics of the more daunting challenges of compound low solar power and low wind energy events. Meanwhile, despite the growing recognition of compound events and their impacts (e.g. drought-heatwave, wind-precipitation and temperature-precipitation extremes), the vulnerability of the renewable energy supply to relevant compound events remains inadequately studied, especially in China, a global major economy and leader in wind and solar energy investments [31]. Recent studies show increasing interest in investigating the spatial disparities of simultaneous LSLW events, although these studies are either only focusing on the historical period at a global scale [32], or on developed countries [18,19], leaving a noticeable gap in comprehending the potential risks of compound LSLW extremes in developing economies, especially within the context of climate change. Additionally, while previous studies have extensively examined climate-induced temporal variability and magnitude changes in wind and solar energy resources, the underlying mechanism governing such shifts remains a critical yet largely underexplored question [5,12,21,33]. A lack of thorough understanding of China's compound LSLW extremes would inevitably hinder power sector planning and a well-informed and proactive renewable energy investment that is necessary for China's carbon neutrality pursuit, which will largely determine the success of global carbon mitigation efforts.

In this study, we present one of the first comprehensive analyses that systematically investigates the spatiotemporal patterns and underlying drivers of compound LSLW extremes over time across China. Historical (1961–1990) and future (2036–2065) climate under SSP126, SSP370 and SSP585 daily meteorological data of five bias-corrected and downscaled CMIP6 general circulation models (GCMs) from the Inter-Sectoral Impact Model Intercomparison Project (ISIMIP) are used in this study. For the historical period, we also provide a sensitivity analysis with the ERA5 data set to enhance the robustness of our results. Specifically, we define compound events for renewable energy as being concurrently below the 10th percentile of daily solar and wind energy resource (i.e. represented by power density: wind or solar power per unit area in this study, W/m^2^) for the period of 1961–1990. Thereby, the frequency of compound renewable events is defined as the average number of days meeting the dual 10th percentile constraints over the study period (e.g. 30 years during 1961–1990), whereas the LSLW intensity is defined as the inverse value of average wind and solar energy resources on identified LSLW days (see Data and Methods). We first evaluate the baseline spatial and seasonal patterns of compound LSLW extremes’ frequency and intensity in response to meteorological variables based on the historical daily variables, and then analyze the impacts of compound LSLW extremes on renewable energy resources across China's regional power grids. Subsequently, we examine the dynamic shifts and robustness of both the frequency and intensity of these compound LSLW extremes under climate change in the middle of the century (2035–2065), unraveling both the statistical patterns and the underlying dynamic mechanisms across various projected climate-socioeconomic scenarios. Moreover, by integrating information on compound LSLW extremes with China's power grid design and interregional electricity transmission plan, we further explore potential strategies to mitigate the impact of compound extremes on renewable energy availability within China's power grids.

## RESULTS

### Spatial patterns of compound low-solar-low-wind extremes

**Figure 1.**
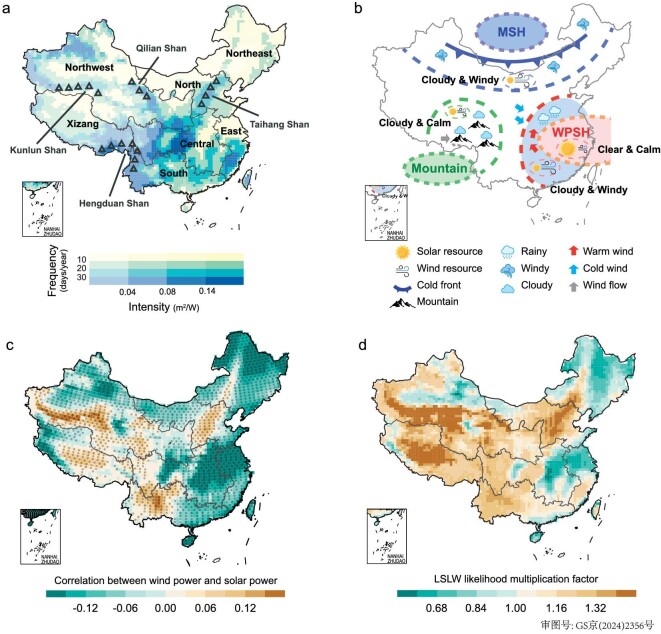
Spatial distribution of historical compound low-solar-low-wind extremes. (a) The spatial distribution of compound LSLW extremes’ frequency (colors) and intensity (shadings) over the historical period (1961–1990) based on multi-model ensemble mean. (b) Schematic diagram of physical processes for inter-diurnal correlation. East Asia is mainly influenced by two weather systems, namely the western Pacific subtropical high (WPSH) and the Mongolian-Siberian high (MSH). In low latitudes, the westward extension of WPSH induces suppressed activity *in situ*, resulting in predominantly clear and calm conditions. Meanwhile, prevailing southerly winds in the west flank of the WPSH transport moisture from the tropics to East Asia, leading to cloudy (low solar) and windy (high wind) conditions. In mid-high latitudes, a cold front invades East Asia under the northerly wind of MSH accompanied by cloudy (low solar) and windy (high wind) weather conditions. In mountainous regions, such as the Tibetan Plateau and Hengduan Mountains, local obstructive effects lead to decreasing wind speed (low wind), and uplift topography with decreasing temperature leads to cloudy conditions (low solar). (c) Inter-diurnal correlation between wind and solar resource based on multi-model ensemble mean. Dotted areas indicate that the correlation coefficients are statistically significant at 95% confidence level based on the student's T-test. (d) Likelihood multiplication factor of our estimated co-occurrences of compound LSLW extremes relative to the counterfactual cases assuming low-solar and low-wind events are independent. The black lines represent China's seven major electricity power grids.

Our results reveal significant spatial heterogeneities in historical annual average frequency and intensity of compound LSLW extremes. Across the country, high frequency compound LSLW extremes are primarily concentrated in southeastern coastal China, eastern Tibetan Plateau, west of the North China Plain and west of Hubei Province, which are predominantly situated near mountain ranges (e.g. Kunlun Mountains, Hengduan Mountains and Taihang Mountains, Fig. 1a), thereby showing evident topographic dependence. Within these regions, compound LSLW events take place 20–30 days/yr, and exceed 30 days/yr in most areas of Yunnan and Sichuan provinces. In contrast, regions characterized by gentle slopes in plain and plateau topography, such as the middle and lower reaches of the Yangtze River Plain, North China Plain, Northeast Plain, the Inner Mongolia Plateau and Western Tibetan Plateau, consistently exhibit a lower frequency of compound events (mostly <10 days/yr), possibly due to generally more stable atmospheric circulation conditions in these areas compared to mountainous regions [4].

The intensity of compound LSLW extremes reflects the severity of wind and solar power deficiencies during LSLW extremes, with a higher intensity indicating diminished renewable energy outputs (see Data and Methods). In contrast to the predominantly geographically driven frequency patterns, the spatial distribution of compound LSLW intensity roughly opposes that of historical annual average renewable energy resources (Figs S2 and S3). Across the country, a high intensity of renewable-energy-related compound LSLW extremes is mainly centered around the Central and East power grids, particularly concentrated in Chongqing-Sichuan, Zhejiang-Fujian and Jiangxi regions (e.g. generally above 0.14 m^2^ W^−1^). Thereby, current hotspots simultaneously exposed to high-frequency-high-intensity LSLW extremes are mainly located in Chongqing and Fujian provinces. Besides, the frequency of compound LSLW extremes shows evident increasing trends across the country, which are particularly strong over southeastern coastal China and southwestern China, yet the spatial distribution of the intensity of compound LSLW extremes’ is generally discrete, with non-significant changing trends (Fig. S4).

Furthermore, to explore the temporal extent of compound LSLW extremes, we further evaluate their average and the annual maximum duration (see Data and Methods, Fig. S5). We observe that both the average duration and the annual maximum duration have spatial patterns similar to those of the frequency of compound LSLW extremes, with the maximum value located in southeastern coastal and southwestern China.

An intriguing correlation emerges between the frequency of compound LSLW extremes and wind-solar power dynamics (Fig. 1b and c). Generally, wind and solar energy are negatively correlated and can compensate for each other. For instance, cloudy and windy weather occurs during the passage of a cold frontal system, and clear and calm conditions occur in southern China under the control of a western Pacific subtropical high [4,28,34] (Fig. 1b). However, wind and solar power can demonstrate strong positive correlations in mountainous regions, as local obstructive effects on wind flow lead to calm conditions and the topography-driven ascending air undergoes adiabatic cooling leading to cloudy conditions. Consequently, there is generally a higher probability of simultaneous wind and solar power deficiencies, and hence higher LSLW frequency in mountainous regions [35,36]. Such an increasing likelihood is notably pronounced across mountainous regions and the likelihood multiplication factor can reach 2 times in southwestern Xinjiang (Kunlun Mountains), and is generally around 1.2–1.5 times in western Sichuan, eastern Tibetan Plateau and Western Yunnan (Hengduan Mountains), as well as in Jing-Jin-Ji regions (Taihang Mountains) (Fig. 1c and d). Therefore, driven by the underlying atmospheric physics, positive wind and solar correlations can largely increase the likelihood of compound LSLW extremes relative to the counterfactual cases assuming wind and solar power are independent (e.g. amplifying by 1.2 times the national average).

### Compromised renewable energy during compound low-solar-low-wind extremes

**Figure 2.**
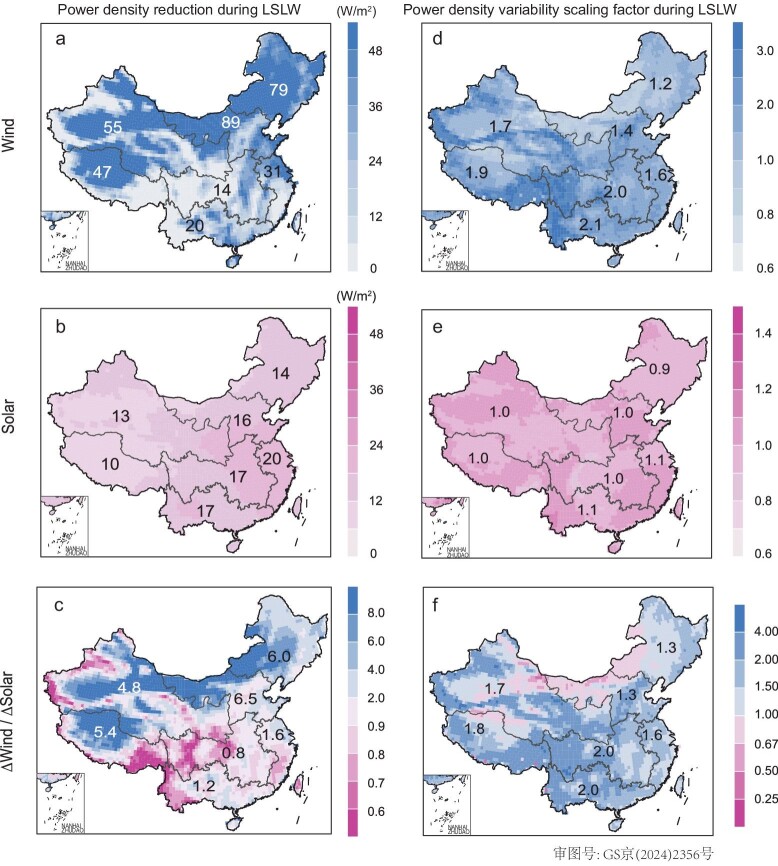
Wind and solar power changes during historical compound LSLW extremes (1961–1990). The spatial distribution of (a) wind and (b) solar resource reduction (power density under average climate minus that under LSLW, RE_average climate_-RE_LSLW_), and the scaling factor of (d) wind and (e) solar resource variability (power density variability under LSLW compared to that under average climate, CV_LSLW_/CV_average climate_), as well as the relative importance between wind and solar in (c) resource reduction ((WE_average climate_-WE_LSLW_)/(PV_average climate_-PV_LSLW_)) and (f) variability changes ((CV_WE LSLW_/CV_WE average climate_)/(CV_PV LSLW_/CV_PV average climate_)) during compound LSLW extremes relative to average climate.

During compound LSLW extremes, both energy resources and the stability of wind and solar power are largely compromised (Fig. 2 and Fig. S3). Both wind and solar resources exhibit noticeable reductions in power density (Δwind: 52.9 W m^−2^, Δsolar: 14.2 W m^−2^) during compound LSLW extremes, with wind power contributing 78.8% of total resource reduction (Fig. S6). As a result, although wind power density often dominates total renewable power density across the country under average climate conditions (e.g. ∼1.6 times solar power density), it only accounts for 26.9% of national solar power density (15.5%–50.1% depending on region) during compound LSLW extremes. In comparison, wind resource variability (e.g. represented by the coefficient of variation of wind power density, CV) is much larger under both an average climate and during compound LSLW extremes (Fig. S7), with wind variability increases mostly dominating variability changes.

**Figure 3.**
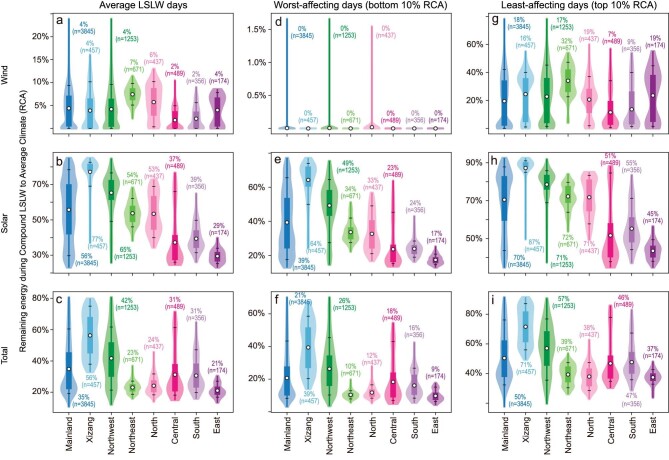
Renewable energy resource deficiency during historical compound LSLW extremes. Violin plots demonstrating country and regional average ratio of the **R**emaining (a) wind, (b) solar and (c) the sum of wind and solar energy resource during **C**ompound LSLW extremes to their respective values under **A**verage climate for the period of 1961–1990 (RCA). (d–f) The bottom 10% worst-affected days with the lowest RCA, and (g–i) the top 10% least-affected days with the highest RCA. The black dots and numbers in violin plots represent the mean value, the upper and lower black lines represent the 90th and 10th percentile grid values within the country or region. n represents the number of grid points within each power grid. Wind energy is largely compromised during compound extremes.

The vulnerability of wind energy during compound LSLW extremes is particularly evident, especially when considering the **R**atio of the remaining wind and solar power during **C**ompound LSLW extremes to their respective daily level 30-yr **A**verage values (**RCA**, Fig. 3 and Fig. S8). For instance, the remaining wind resources are on average merely 4.4% of those under an average climate (ranging from 1.8% and 2.1% in the Central and South power grids to 7.4% in the Northeast power grid). National average RCA for wind energy can even reduce to 0.004% on worst-affected days (the bottom 10% of days with the lowest RCA), with the RCA being ∼18.3% on the least-affected days. Solar resources demonstrate much larger remaining energy availability and different spatial patterns in comparison to wind power. The national average solar RCA is ∼55.7% of that under an average climate (ranging from 29.5% in the East power grid to 77.2% in the Xizang power grid). Even on the worst-affected days, the national average RCA for solar power is ∼39.4%, compared with 70.4% on the least-affected days; much larger than that for wind RCA.

The national average RCA for the sum of solar and wind power is ∼34.8% (20.7% and 50.4% on the worst- and least-affected days, respectively), ranging from ∼20% in eastern China (e.g. East, North and Northeast power grids) to over 40% in western China (e.g. Xizang and Northwest power grids). Consequently, renewable energy resource declines during compound LSLW extremes are primarily dominated by wind power decreases, and the relative energy declines in eastern China are particularly impacted during compound LSLW extremes compared to normal days.

### Climate-induced changes in compound low-solar-low-wind extremes

**Figure 4.**
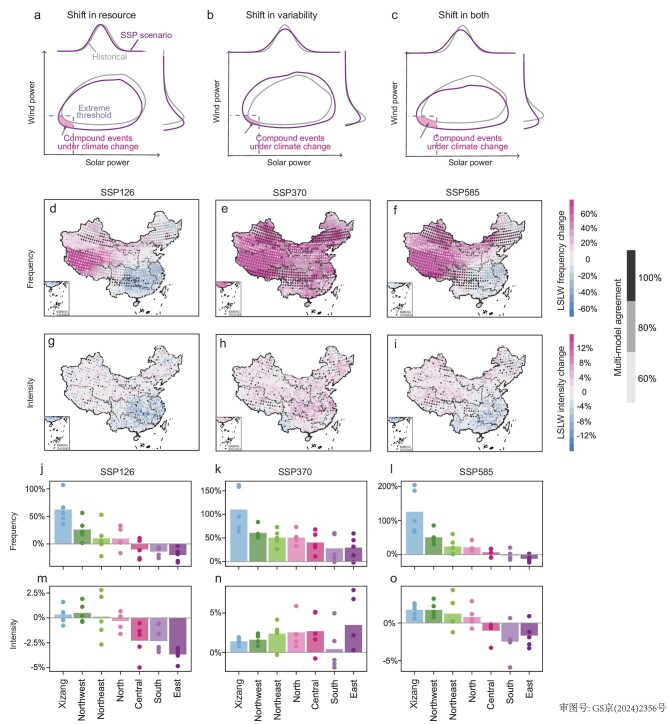
Climate change effects on compound LSLW extremes’ frequency and intensity. Changes in the probability of compound LSLW extremes (shift of the bivariate distribution) arising from (a) a shift in the average of wind and solar resources, (b) a shift in the variability of wind and solar resources and (c) shifts in both the average and variability of wind and solar resources. Dashed lines indicate the LSLW extreme threshold. Percentage changes (%) in the (d–f) frequency and (g–i) intensity of compound LSLW extremes under (d, g) SSP126, (e, h) SSP370 and (f, i) SSP585 scenarios over 2036–2065 relative to the historical period (1961–1990). Gray shading points denote that more than 60% of models agree on the sign of change. Regional-average changes (%) in the (j–l) frequency and (m–o) intensity of compound LSLW extremes under (j, m) SSP126, (k, n) SSP370 and (l, o) SSP585 scenarios over 2036–2065 relative to the historical period (1961–1990). The points are individual model values and the bars represent the mean values of five climate models.

Future climate change could reshape the spatial and temporal dynamics of compound LSLW extremes’ frequency and intensity via affecting wind and solar resources and variability (Fig. 4 and Fig. S9) [37,38]. As shown in Fig. 4, frequency maps illustrate evident and significant increases across western China under climate change, with substantial and consistent frequency rises observed particularly under SSP370. National average frequency increases are 60.2% under SSP370—4.86 and 1.60 times those under SSP126 and SSP585, respectively. Across the country, Xizang experiences the largest frequency increases under all scenarios, ranging from 61.1% (12.0 days/yr) under SSP126, to 107.3% (21.1 days/yr) and 121.7% (23.9 days/yr) under SSP370 and SSP585, respectively (Fig. 4j–l and Fig. S9g–i). Whereas all seven power grids experience net compound LSLW frequency increases across the country under SSP370, the Central, East and South power grids in eastern parts of the country are exposed to small yet non-negligible decreases under SSP126, with even smaller frequency decreases observed in eastern China under SSP585 (Table S1). The relative and absolute compound LSLW frequency and intensity changes demonstrate a similar spatial pattern under the same climate scenario (Fig. 4 and Fig. S9). Besides, changes in compound LSLW average duration and annual maximum duration are also considerably greater across the country, especially in Xizang (Figs S10 and S11).

Notably, the increasing frequency of compound LSLW extremes under climate change is linked to both notable renewable resource declines and their increasing variability (Fig. 4a–c). For instance, as illustrated in Fig. S9m, under both magnitude reductions and more tailed distributions for wind and solar power (e.g. primarily due to wind speed and solar radiation as shown in Figs S12–S14), renewable energy resources on increasing days may fall below the dual 10th percentile LSLW thresholds.

Here we further explore the underlying mechanisms for the projected changes in compound LSLW extremes across scenarios, focusing on declines in wind speed and solar radiation (Figs S15). Wind is essentially created by pressure gradients associated with uneven heating of the Earth's surface [39]. Over mid-latitude northern China, future warming weakens this driving force (i.e. pressure-gradient force) by diminishing the meridional temperature gradient, and ultimately reduces surface wind speed. Similar mechanisms have also been identified for historical global terrestrial stilling [9,40,41], and the influence grows more pronounced with enhanced warming (Figs S16 and S17). Concurrently, the increased frequency of extremely low surface solar radiation aligns with the overall decline in surface solar radiation, primarily driven by the increased frequency of extremely dense cloud cover (Figs S18). It is noteworthy that the distinct increase in aerosol loading in SSP370, compared to the other two scenarios, likely plays an important role in driving the most striking increases in LSLW frequency observed nationwide. The high aerosol loading weakens wind speed by inducing a more stable and lower boundary layer and attenuates radiation both directly, by absorbing and scattering incoming solar radiation, and indirectly, by modifying cloud lifetime (e.g. increasing cloud albedo through Twomey effect and prolonging cloud lifetime) [42,43]. In comparison, a slight decline in the frequency of LSLW is found over southern China, especially in summer, in SSP126 and SSP585, along with increasing wind speed and solar radiation (Fig. 4d–f). The amplification of wind speed can be attributed to an enhanced East Asian summer monsoon, indicated by the intensified land–sea pressure gradient due to faster warming of land than ocean, while the increase in surface solar radiation is associated with decreased aerosol loading and cloud cover (Figs S18 and S19).

Intensity maps show less pronounced changes than frequency maps, with the most significant increases observed, yet again, under SSP370, notably in the Central (3.3%) and East (3.2%) power grids (Fig. 4g–i and Fig. S9d–f). The intensity of compound LSLW extremes is generally increasing across all power grids under SSP370 (2.1%), compared with slight increases under SSP585 (0.4%), and mostly decreases across the country under SSP126. Overall, intensity changes are much less robust than frequency changes, that is, with far fewer cross-model agreement than frequency changes under all SSP scenarios evaluated here. Likewise, RCA values under climate change are only slightly different from the historical values (e.g. mostly within a 4-percentage-point difference, Figs S20–S22). Therefore, future renewable energy planning should be particularly concerned with more frequent simultaneous LSLW power shortages, though the resulting energy deficiencies during compound events are more or less the same as that under the historical period. Seasonal changing patterns generally resemble that of annual changes (Figs S23 and S24).

### Power grid vulnerability under compound low-solar-low-wind extremes

In fulfilling China's carbon neutrality commitment, China needs to significantly increase its solar and wind power generation to substitute fossil electricity. Therefore, there are strong motivations for taking advantage of solar and wind power in resource-rich regions.

**Figure 5.**
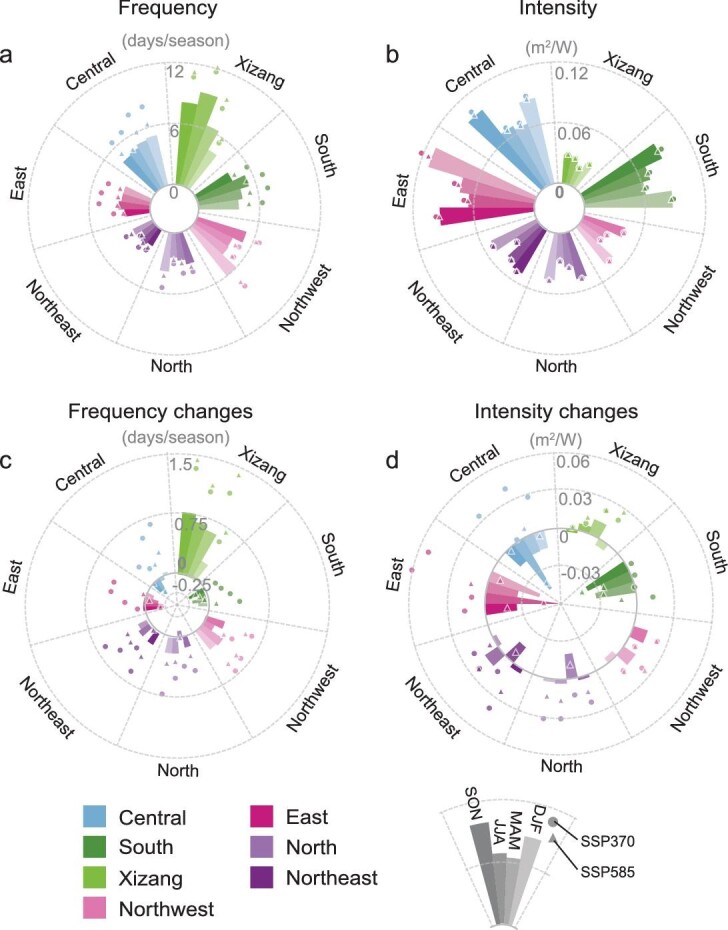
Regional compound LSLW extremes’ frequency and intensity. The (a, c) frequency and (b, d) intensity and their respective absolute changes across China's seven major power grids under SSP126 (histogram), SSP370 (circle) and SSP585 (triangle) scenarios across four seasons.

In China's 14th Five-Year Plan, total planning installation capacity for wind energy and solar PV in the North, Northeast and Northwest power grids ranks among the top three of China's electricity power grids (circle sizes in Fig. S25). These three power grids, especially the North and Northeast grids, generally have the most abundant solar and wind resources under climate change, and often with relatively low frequency and intensity of compound LSLW extremes, making these regions relatively good candidates for wind and solar power development (Fig. 5 and Figs S25 and S26).

In comparison, the South and Central power grids have the lowest renewable resources and still suffer from almost the most frequent and intensified compound LSLW extremes under climate change, despite overall decreases, rendering these regions among the least favorable for wind and solar deployment (Fig. 5 and Figs S25 and S26). Renewable energy power density and variability in the East power grid are mostly middle ranking, along with having high LSLW intensity, which may lead to severe power deficiencies, particularly in winter. Xizang, with the least progressive renewables development plan in China's 14th Five-Year Plan, also often has a middle-ranked power density and the lowest LSLW intensity, yet it has among the most frequent compound LSLW extremes under climate change. Thereby, all power grids are exposed to a certain number of LSLW-induced high renewable power shortages, which will further increase under a warming climate (particularly SSP370 and SSP585), highlighting the importance of adaptative power grid planning.

### Effective climate-permitting interregional electricity transmission

With strong heterogeneous climate impacts on wind speed and solar radiation across China, interregional renewable energy transmission, which is contingent upon favorable meteorological conditions, can be an important adaptation strategy to tackle more frequent compound LSLW extremes.

**Figure 6.**
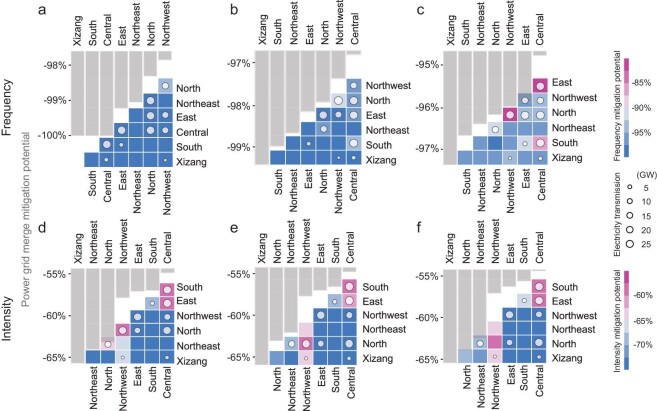
Regional compound LSLW extremes’ frequency and intensity mitigation potential. The mitigation potential for the (a–c) frequency (d–f) and intensity of compound extremes via interregional electricity transmission under SSP126. (a, d) 5th percentile thresholds for compound LSLW extremes, (b, e) 10th percentile thresholds for compound LSLW extremes, and (c, f) 20th percentile thresholds for compound LSLW extremes. Circles in subpanels indicate the actual amounts of current interregional electricity transmission in 2021. Gray bar shows the average mitigation potential (AMP) of interregional electricity transmission for each region in connecting with all other regions.

Previous studies find that aggregating wind and solar resources over large areas could reduce the resource variability and the spatial-temporal imbalance between renewable energy availability and power demand can be alleviated via grid connection [44,45]. Liu *et al.* demonstrate that interregional grid connection is the most promising strategy to integrate variable renewable energy into China's power system, highlighting the remarkable potential of grid connection for improving renewable power penetration and reducing renewable power variability [44]. Here, we explore whether, and to what extent, grid connection for renewables can mitigate the frequency and intensity of compound LSLW extremes (Fig. 6). Interregional electricity transmission is particularly effective in mitigating both the frequency of compound LSLW extremes (by 91.1%–99.7%) and their intensity (by 58.7%–84.8%). Reduction potential generally demonstrates larger mitigation potential for stricter compound extremes (5th percentile thresholds) than looser (20th percentile thresholds) ones. Despite the magnitude differences, the overall mitigation patterns are more or less consistent. In particular, grid connection between the Xizang and other power grids demonstrates the most promising mitigation potential with regard to both LSLW frequency and intensity, generally followed by the South, Northeast and East power grids in terms of frequency reduction, and followed by the North, Northeast and Northwest power grids in terms of intensity alleviation.

That said, lower mitigation potential often occurs in provinces in close proximity or within the same regional power grid, particularly for the transmission from Hebei to Beijing or to Tianjin within the North power grid, which achieves merely 30% and 45% of frequency reductions, respectively (Fig. S27). This is probably because, as mentioned before, unlike fossil-based electricity, interregional renewable electricity transmission is contingent upon favorable meteorological conditions (i.e. climate permitting), thus connection between distant regions that may exhibit distinct weather systems is more effective in reducing the likelihood of compound LSLW extremes, and therein facilitates a more reliable renewable energy transmission. Energy storage may thus play a more important role in such cases. However, current interregional electricity transmission infrastructure is largely insufficient in these regions, especially in Xizang, due to both geographical and economic challenges in developing (ultra-) high-voltage electricity transmission.

## DISCUSSION

Achieving a rapid low-carbon energy transition to mitigate climate change requires progressive wind and solar energy development. Yet, these renewable sources often face criticism for their variability, intermittency and unpredictability. Compound LSLW extremes in particular pose a severe threat to reliable renewable energy availability—threat that may intensify with climate change. Therefore, our study represents one of the first efforts to systematically analyze the spatiotemporal patterns and the underlying drivers of compound LSLW extremes over time across China. These insights are pivotal in facilitating the low-carbon energy transition and supporting China's pursuit of carbon neutrality.

Across the country, hotspots of high-frequency and high-intensity compound LSLW extremes are primarily concentrated in the Central and South power grids. These areas, which also have the lowest wind and solar energy resources, present significant challenges for local renewable energy development, despite being key energy demand centers in China. As cost-effective wind and solar power stations are planned for installation near these major load centers [44,46], regions that already have low renewable energy potential will face increasing difficulties in developing local wind and solar power due to severe compound LSLW extremes. Conversely, while the Northwest, Northeast and North power grids generally have the highest renewable energy resources and experience the least frequent and intense LSLW, they are exposed to notable increases in LSLW frequency under a warming climate; thus they are also not immune to LSLW risks (Figs S3 and S25).

Effective adaptation strategies are imperative for China's major power grids facing different levels of compound LSLW extremes. We find that inter-grid electricity transmission emerges as a promising solution, significantly reducing the potential impacts of compound LSLW extremes on energy output failures, decreasing their frequency by over 91% and intensity by 59%–85%. Notably, Xizang often demonstrates the largest potential in mitigating LSLW-induced renewable power shortages for other regions via grid interconnection. In recent years, with the increase in the installed capacity of wind and solar power in Xizang and northern China, renewable energy cannot be fully absorbed due to small local electricity consumption; poor interregional electricity grid connection between non-energy-demand centers and energy-demand centers leads to high abandonment rates of solar and wind energy [44]. In response to current constraints, development plans are either targeting expanding energy demand to Xizang and northern China (e.g. by relocating energy-consuming industries or developing local energy industries such as hydrogen) or developing (ultra-)high-voltage electricity transmission lines to export renewable power in Xizang and northern China to consumption centers in Central and East China, to better facilitate China's carbon neutrality targets. Enhanced interregional electricity transmission, particularly involving Xizang generally followed by the South and Northeast (North and Northeast) power grids, exhibits effectiveness in mitigating the frequency (intensity) of compound LSLW extremes across regions, highlighting its potential in ensuring reliable renewable electricity availability through grid development along with other energy technologies (e.g. energy storage).

Climate change can lead to increasingly frequent yet only slightly more intensified compound LSLW extremes across Xizang and northern China (e.g. the Northwest, North and Northeast power grids). In particular, we observe the most substantial and robust LSLW frequency (60.2%) and intensity (2.1%) increases across China under SSP370 due to its largest resource reduction combined with the most striking tailed meteorology distributions, and therein the largest variability increases for wind and solar power (Figs S28–S30). LSLW increases, especially for frequency, are less substantial, but are still notable under SSP126 (12.4%) and SSP585 (37.6%), respectively. Increasingly frequent and intensified LSLW under climate change is primarily driven by the weakened meridional temperature (pressure) gradient and increased frequency of extremely dense cloud cover with the additional distinctive influence of increased aerosols under SSP370. Our findings highlight that the escalating frequency and intensity of compound extremes under climate change could present significant challenges to reliable wind and solar energy resources, posing uncertainties that could complicate energy planning and impede energy sector investments. Importantly, by elucidating the underlying mechanisms, we emphasize that such climate-induced compound LSLW extreme changes are not simply by chance, but rather largely foreseeable with the help of atmospheric physics, underscoring the possibility for proactive preparation and mitigation measures.

Our study is subject to several limitations. First, our analysis relies on multi-model ensemble mean meteorological variables to estimate the frequency and intensity of compound LSLW extremes. This approach ensures a fair and consistent comparison between the historical period and future climate. To characterize the uncertainties in our estimated historical compound LSLW extremes, we evaluate the robustness of our results through multi-model comparisons, and find that the spatial pattern for the frequency and intensity of compound LSLW extremes, based on the ISIMIP data set, exhibits satisfactory spatial consistency with the ERA5 reanalysis data set from 1961–1990 (with the spatial correlation ranging from 0.93–0.95 for frequency and 0.58–0.64 for intensity, Fig. S31). However, our estimated compound LSLW extremes are generally conservative compared to ERA5 results (Fig. S32), indicating potentially even more severe compound LSLW extremes in the real world. Moreover, the ISIMIP data set also captures the spatial patterns of wind and solar power density reduction. However, the magnitude of the decline in wind power density is greater than that observed in the ERA5 data set (Fig. S33), potentially leading to slight overestimations of the contribution of wind power reductions. Second, significant uncertainties exist with regard to the future climate and socioeconomic changes. To address this, we evaluate a series of shared socioeconomic pathways (e.g. SSP126, SSP370 and SSP585). This approach offers a relatively comprehensive coverage of future climate changes, using multi-model ensemble means for each scenario and its cross-model agreement to characterize compound LSLW extremes and their uncertainties under climate change. Third, the relatively coarse resolution of global climate models may not capture finer-scale physics. For example, the China Wind and Solar Energy Resource Bulletin reports ridge wind resources in mountainous regions (e.g. Yunnan-Guizhou Plateau and the Tibetan Plateau) based on historical high resolution (1 km × 1 km) wind and solar resource compilation, which, however, does not provide corresponding data under climate scenarios [47]. While our study relies on history-future consistent multi-model simulations to represent LSLW characteristics, future development of finer resolution data sets spanning the entire historical and future timeframe will be needed to capture finer-scale features. Finally, we define the frequency and intensity of compound LSLW extremes based on the 10th percentile thresholds of daily wind and solar power over the 30-yr study period. However, this approach might not fully account for inter-diurnal variations. To address this, we use a 15-day rolling window of solar PV and wind energy over 30 years to form data sets of 450 values for each day, enhancing the stability of the probability distribution of wind and solar power (see Data and Methods). In addition, we use different thresholds (e.g. 5%, 10% and 20%), which show similar results to the main analysis based on the 10th percentile thresholds. For instance, with a lower compound LSLW extremes threshold (5th percentile), the spatial distribution of frequency and intensity is generally the same as under the 10th percentile, albeit with lower values given a lower threshold applied (Fig. S34). Compared to the 10th percentile thresholds, the spatial patterns of LSLW frequency and intensity changes under corresponding SSP scenarios are similar, although frequency increases under the 5th percentile thresholds are larger. For instance, national average LSLW frequency increases are 23.7%, 84.2% and 58.3% under SSP126, SSP370 and SSP585, respectively, with the 5th percentile thresholds (Fig. S35), exceeding the corresponding values of 12.4%, 60.2% and 37.6% under each SSP with the 10th percentile thresholds, indicating even more substantial increases in extremely low renewable energy resources under a warming climate.

By revealing the geospatial and temporal evolution of the frequency and intensity of China's compound events, along with the underlying mechanisms, our study sheds light on the previously overlooked yet daunting compound LSLW extremes. Our results underscore the importance, for nations engaging in progressive decarbonization, of factoring compound LSLW extremes into renewable energy development and power sector planning. Doing so will facilitate a more robust low-carbon energy transition and aid in the pursuit of carbon neutrality.

## DATA AND METHODS

### ISIMIP climate input data sets

In order to get more detailed results over China, we collected five bias-corrected and downscaled CMIP6 GCMs from the ISIMIP framework (https://www.isimip.org): GFDL-ESM4, IPSL-CM6A-LR, MPI-ESM1-2-HR, MRI-ESM2-0 and UKESM1-0-LL at a spatial resolution of 0.5° × 0.5° for daily meteorological variables.

The bias-adjustment method employs a quantile mapping approach and relies on the observational W5E5 v.1.0 data set [48]. As explained in the ISIMIP documentation [49], the GCM selection is based on data availability at the time of selection, performance in the historical period, structural independence, process representation and equilibrium climate sensitivity (ECS). The five GCMs are structurally independent in terms of their ocean and atmosphere model components and overall they represent the range of ECS across the full CMIP6 ensemble.

The daily weather variables at 0.5° spatial resolution that are computed for wind energy and solar PV output include: daily mean downwelling shortwave irradiance (rsds, W/m^2^), daily mean surface pressure (ps, hPa), daily mean surface wind speed (sfcWind, m/s) and near-surface air temperature (tas, K).

Further, we employ a variety of variables to diagnose the large-scale circulation pattern associated with compound LSLW extremes. To analyze the circulation patterns, we use daily data from the CMIP6 model in accordance with the aforementioned five models, including sea-level pressure (SLP, Pa), fraction of total cloud cover (clt, %), aerosol optical depth at 550 nm (od550aer), and zonal and meridional wind at 850 hPa (ua and va, m/s). All model variables are remapped to 1° spatial resolution.

### Compound low-solar-low-wind extremes

Using the above-calculated daily average grid-level (0.5° × 0.5°) wind and solar power resources for both the historical period (1961–1990) and the future climate scenario (2036–2065), we define a compound LSLW extreme event as a condition where both wind energy and solar PV power simultaneously fall below their respective historical daily 10th percentile values (see details in Supplementary Data). The threshold values for low wind and solar extremes are obtained from the baseline historical period (1961–1990) and applied in the future climate (2036–2065), such that we can track the LSLW frequency and intensity changes under a warming climate in comparison to the historical period.

Following the methodology widely used for assessing changes in the probability of the occurrence of extreme events, we consider the extreme thresholds in both temporal and spatial aspects. Firstly, for each grid, we use a 15-day rolling window of the solar PV and wind energy over the baseline historical period of 30 years (1961–1990) to form data sets of 450 values for each day (e.g. 450 values for 1 January). At each grid point of a climate model, the data set mean represents a daily level climatology. Such rolling average daily percentiles not only provide a more robust probability density function (PDF) for the reference period (e.g. giving less weight to outliers on a particular day), but also act to distinguish especially intense events from more typical cases within one year. Here we specifically use grid-level threshold to account for both geographical variations in local wind and solar energy resources and the location-specific extreme low energy resources that relate to the intermittency of wind and solar energy. The location-specific 10th percentile threshold for wind and solar energy resource is shown in Fig. S36.

The frequency of compound LSLW extreme events is the number of days meeting the dual 10th percentile constraints averaged over 30 years (e.g. days per year). The intensity of a compound LSLW event is measured by the inverse of the averaged sum of wind and solar resources occurring on LSLW days. Thus, the lower the renewable energy output during the extreme event, the stronger the intensity. As the amount of wind and solar resources across the country is comparable (5.65 W/m^2^ to 63.79 W/m^2^, varying by 11.25 times), the compound LSLW extremes’ intensity variations are also not too skewed (0.016 m^2^/W to 0.180 m^2^/W). In addition, we use two metrics to illustrate the duration of compound LSLW extremes: ‘average duration’ represents the average number of continuous days when a compound LSLW event occurs, and ‘annual maximum duration’ represents the longest duration of compound LSLW extremes in a year for both the historical period and the future climate (Figs S5 and S37). We then further demonstrate the dynamic mechanisms of compound LSLW extremes via attribution analysis.

We split the whole country into seven regional power grids: Northeast, North, Northwest, Xizang, Central, East and South (Fig. S1). Regional average frequency and intensity of compound LSLW extreme events is estimated by averaging area grid values.

## Supplementary Material

nwae424_Supplemental_File

## Data Availability

Data used to perform this work can be found in the Supplementary Data. Numerical results for Figs 1–6 will be provided with this paper as source data. Any further data that support the main findings of this study are available from the corresponding author upon request.
