# Supplementary material for: Unraveling climate change-induced compound low-solar-low-wind extremes in China
Source: Natl Sci Rev. 2024 Nov 25;12(1):nwae424. doi: 10.1093/nsr/nwae424 (PMC11715666; doi:10.1093/nsr/nwae424)
Supplement: nwae424_Supplemental_File [file nwae424_supplemental_file.docx]

**Supplementary Materials for**

**Unraveling climate change-induced compound low-solar-low-wind extremes in China**

Licheng Wang^1,2,†^, Yawen Liu^3,†^, Lei Zhao^4,5^, Xi Lu^6^, Liangdian Huang^1,2^, Yana Jin^1,2^, Steven J. Davis^7^, Amir Aghakouchak^7,8^, Xin Huang^3^, Tong Zhu^1,2,*^, Yue Qin^1,2,*^

*^1^ College of Environmental Sciences and Engineering, Peking University, Beijing, 100871, China*

*^2^ Institute of Carbon Neutrality, Peking University, Beijing, 100871, China*

*^3^ School of Atmospheric Sciences, Nanjing University, Nanjing, 210023, China*

*^4^ Department of Civil and Environmental Engineering, University of Illinois at Urbana-Champaign, Urbana, IL 61801, USA*

*^5^ National Center for Supercomputing Applications, University of Illinois at Urbana-Champaign, Urbana, IL 61801, USA*

*^6^ School of Environment, State Key Joint Laboratory of Environment Simulation and Pollution Control, Tsinghua University, Beijing 100084, China*

*^7^ Department of Earth System Science, University of California, Irvine, Irvine, CA 92697, USA*

*^8^ Department of Civil and Environmental Engineering, University of California, Irvine, Irvine, CA 92697, USA*

*Correspondence to: [qinyue@pku.edu.cn](mailto:qinyue@pku.edu.cn); [tzhu@pku.edu.cn](mailto:tzhu@pku.edu.cn)

^†^Equally contributed to this work.

**Supplementary methods**

**Calculation of wind energy resource**

Wind energy resource is mostly a function of the instantaneous wind speed. When wind speed is higher than the cut-in wind speed, and lower than the speed required for the nameplate capacity, instantaneous wind energy density (WE, W m^-2^) is estimated as below (Eq. 1):

$WE=\frac{1}{2} \rho{ws}^{3}$ (1)

Where $\rho$ presents the air density, which is assumed to be a constant value of 1.225 kg/m^3^ at standard atmospheric conditions, $ws$ (m/s) represents the wind speed at the hub height of wind turbine.

Wind energy density remains constant after reaching the nameplate capacity until the wind turbine is shut down when the wind speed is too high. Following earlier studies [1,2], the constants 3 m s^-1^, 12 m s^-1^ and 25 m s^-1^ are assumed as cut-in wind speed, nameplate wind speed, and cut-out wind speed, respectively. Therefore, the full relationship between the instantaneous wind speed and the instantaneous wind energy density is given by the following Eq. 2:

$WE=\left\{ \begin{aligned} 0, ws<3 m s^{-1} or ws>25 m s^{-1} \\ \frac{1}{2} \rho{ws}_{100}^{3} , 3m s^{-1} < ws< 12 m s^{-1} \\ 1058.4, 12 m s^{-1} \leq ws\leq25 m s^{-1} \end{aligned} \right.$ (2)

We use the wind speed at 100m height above the surface to represent the wind speed across the rotor plane. It is noted that wind speed at 100m is not available from climate model outputs here. Similar to previous studies [1,3], wind speed at 100m is approximately extrapolated from the wind speed at 10m height using the power law:

${ws}_{100}={ws}_{10}{(\frac{100}{10})}^{\alpha}$ (3)

Here, ${ws}_{10}$ is the daily mean wind speed at 10 m extracted from ISIMIP datasets. The scaling factor of $\alpha$, representing how quickly the wind decays towards the ground, is often approximated as a constant of 0.143 over land surface in previous studies [1,3].

**Calculation of solar PV resource**

Solar power density primarily depends on solar irradiance, and to a lesser extent, ambient temperature and wind speed via affecting power output efficiency. Following previous studies [1,3–5], we take solar radiance ($I,$W m^-2^), surface temperature ($T$, °C), and wind speed ($WS$, m s^-1^) as input parameters and simulate solar resource per 1 m^2^ of solar panel (PV, W m^-2^):

$PV=I\times\mu_{pv}$ (4)

where $I$ is the solar radiance on the PV module per m^2^ (W m^-2^); $\mu_{pv}$ is the efficiency of the PV module, which is determined by weather conditions. $\mu_{pv}$ is estimated via Eq. 5:

$\mu_{pv}=\mu_{panel}\times(1+\gamma\times(T_{panel}-T_{STC}))$ (5)

where $\mu_{panel}$ is the assumed panel efficiency under standard conditions (17%); $\gamma$ is the typical efficacy response of monocrystalline silicon solar panels (-0.005 °C^-1^); $T_{STC}$ is the panel temperature under standard conditions (25°C); and $T_{panel}$ is the panel temperature determined by temperature, irradiation and wind speed:

$T_{panel}=c1+c2\times T+c3\times I+c4\times WS$ (6)

where $c1$ is 4.3°C; $c2$ is 0.943; $T$ is surface temperature (°C); $I$ is the solar radiance on the PV module (W m^-2^); $c3$ is 0.028 °C m^2^ W^-1^, $c4$ is -1.528 °C s m^-1^, and $WS$ is the surface wind speed (m s^-1^). Therefore, when $T_{panel}$ surpass $T_{STC}$, the declined panel efficiency would further reduce solar PV power density. These coefficients represent the influence of meteorological conditions on the cell temperature. The ambient $T$ determines the base temperature of the cell, a strong $I$ increases the cell temperature and $WS$ decreases cell temperature.

Both wind and solar power are heavily influenced by weather fluctuations, hence yields strong daily variability. We use the metric of coefficient of variation (CV) to quantify the day-to-day variability of wind and solar resource using Eq. 7.

$CV=\frac{Standard deviation (resource)}{mean (resource)}$ (7)

**Calculation of RCA**

In order to illustrate the impacts of resource under compound low-solar-low-wind (LSLW) extremes on climatology condition, here we introduce a metric named the **R**atio of **C**ompound to **A**verage (RCA), which represents the ratio of remaining wind or/and solar power during compound LSLW extremes to their daily-level 30-year average values. Firstly, we calculate the long-term climatology (1961-1990, baseline) of wind and solar resource at each grid point. Secondly, we select days with compound LSLW extremes and then calculate their average values of the remaining wind and solar resource. The RCA is obtained by dividing the remaining wind and solar resource by long-term climatology average. Further, we rank the remaining wind and solar resource during compound LSLW extremes, defining the top 10% days (90^th^ percentile RCA) as the least-affecting days, while considering the bottom 10% days (10^th^ percentile RCA) as the worst-affecting days.

**Mitigation potential of inter-grid electricity transmission**

In order to demonstrate the effectiveness of grid interconnection in mitigating compound LSLW extremes’ frequency and intensity, we employ two approaches here to quantitatively assess its mitigation potential. First, we consider the region (power grid or province) as a whole, and calculate the regional average daily wind and solar energy resource. At each region, we count the date of regional compound LSLW extremes using the quantile threshold method (below the 10^th^ percentile daily solar and wind energy resource respectively). Subsequently, considering a day when a compound LSLW extreme occurs in region A but not in region B, it is assumed that renewable energy can be transmitted from region B to compensate for LSLW-induced energy supply declines in region A. Total number of compound LSLW extreme days in region A is $d1$, and the number of days mitigated by region B is $d2$, so the un-mitigated days in region A are $d1-d2$. Therefore, the mitigation potential (%) for region A’s LSLW frequency via integrating with region B can be calculated by Eq. 8:

$frequency mitigation potential=\frac{d1-d2}{d1}$ (8)

Similarly, considering a day when a compound LSLW extreme occurs in region A but not in region B, we calculate the respective resource magnitude in region A ($r1,W m^{-2}$) and region B ($r2,W m^{-2}$) on that day. Correspondingly, the intensity of compound LSLW extremes is represented by $\frac{1}{r1}(m^{2} W^{-1})$ and $\frac{1}{r2}(m^{2} W^{-1})$, respectively. We also assume that renewable energy can be transmitted from region B to compensate for LSLW-induced energy supply declines in region A. Total resource available for inter-connection is denoted as $r1+r2$ (with intensity expressed as $\frac{1}{r1+r2}$). Therefore, the LSLW intensity mitigation potential (%) for region A via integrating with region B can be expressed in Eq. 9:

$intensity mitigation potential= \frac{\frac{1}{r1+r2}-\frac{1}{r1}}{\frac{1}{r1}}$ (9)

Besides, in order to evaluate the overall effectiveness in region B’s mitigating potential for all other power grids, we calculate the average value of the frequency (or intensity) mitigation potential when connecting region B with each individual power grid, which is defined as the **A**verage value of the **M**itigation **P**otential (AMP) (%) for LSLW frequency (or intensity) of region B, as represented by the gray bar in Fig.6 and Supplementary Fig. S22.

**Calculation of Likelihood multiplication factor**

We use the **L**ikelihood **M**ultiplication **F**actor (LMF) to illustrate the impact of the possible correlation between wind and solar energy resources on their joint occurrence probability [6]. LMF is calculated as estimated co-occurrences of compound low-solar-low-wind extremes (P_actual_) relative to the counterfactual cases assuming low-solar and low-wind events are independent (P_indep_). The LMF_i_ (Eq.10) is determined for each grid cell individually by calculating grid cell-specific P_actual,i_ (Eq. 11) and P_indep,i_ (Eq.12) as:

$\mathrm{LMF}_{i}=\frac{P_{actual,i}}{P_{indep,i}}$ (10)

$P_{actual,i}=\frac{\sum low\_solar\_low\_wind}{\mathrm{ndays}}$ (11)

$P_{indep,i}=\frac{\sum low\_solar}{\mathrm{ndays}}\times\frac{\sum low\_wind}{\mathrm{ndays}}$ (12)

where, ndays refers to total days in the historical study period (1961-1990) for 10958 days, and future period (2036-2065) for 10957 days. $\sum low\_solar$ and $\sum low\_wind$ refer to the days falling below their respective 10% threshold. And $\sum low\_solar\_low\_wind$ refer to the compound LSLW extremes’ days.


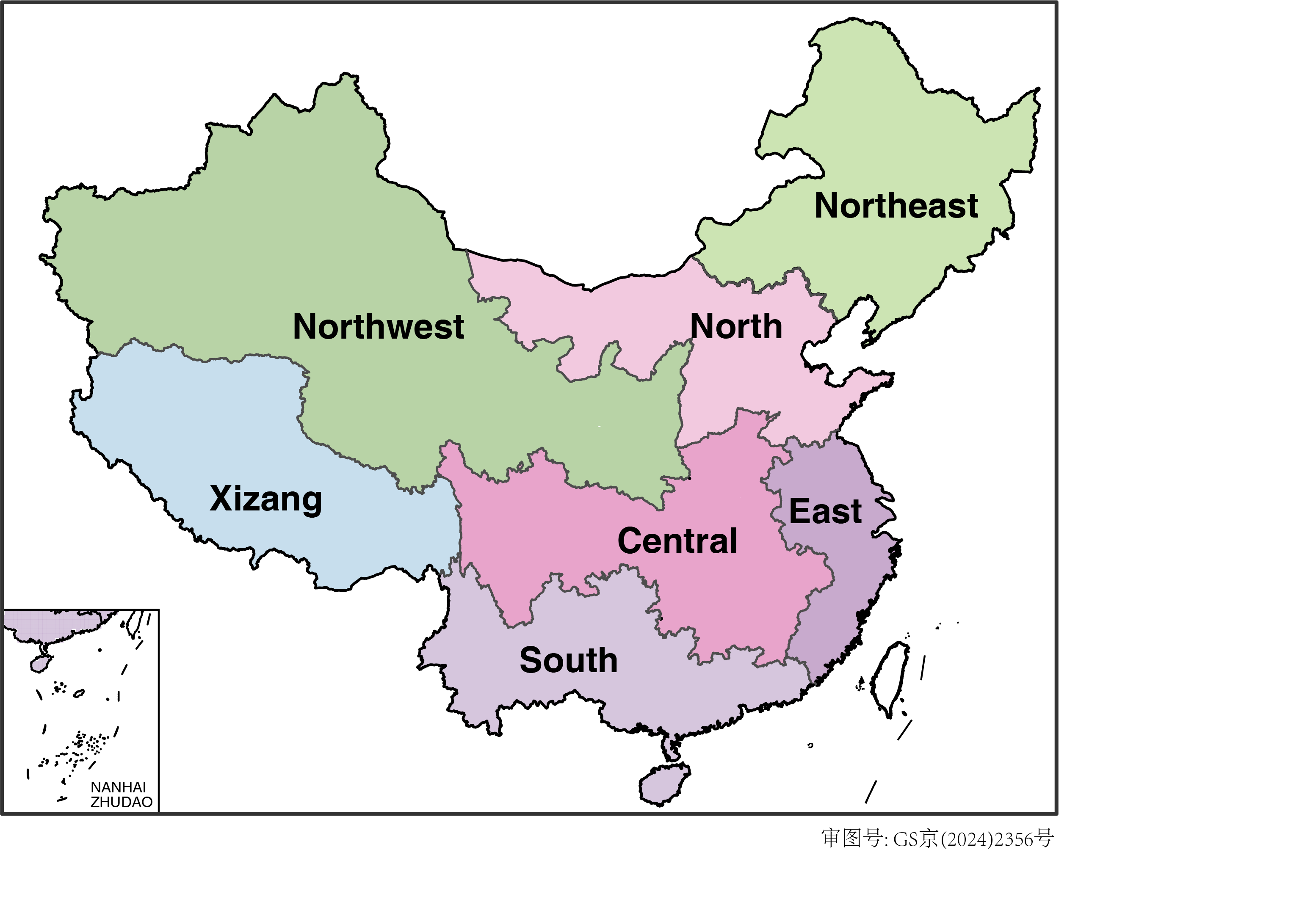


**Supplementary Figure S1.** Spatial distribution of China’s major electricity power grids. The seven regional major electricity power grids include: the Northeast power grid (e.g., Heilongjiang, Jinlin, Liaoning, and east Inner Mongolia), the North power grid (e.g., Beijing, Tianjin, Hebei, Shandong, Shanxi, and wast Inner Mongolia), the East power grid (e.g., Jiangsu, Anhui, Shanghai, Zhejiang, and Fujian), the Northwest power grid (e.g., Xinjiang, Qinghai, Gansu, Ningxia, and Shaanxi), the Central power grid (e.g., Henan, Hubei, Sichuan, Chongqing, Hunan, and Jiangxi), the South power grid (e.g., Guangxi, Guizhou, Guangdong, and Hainan), and the Xizang power grid. No data available for Taiwan, Hong Kong, and Macao in this study.


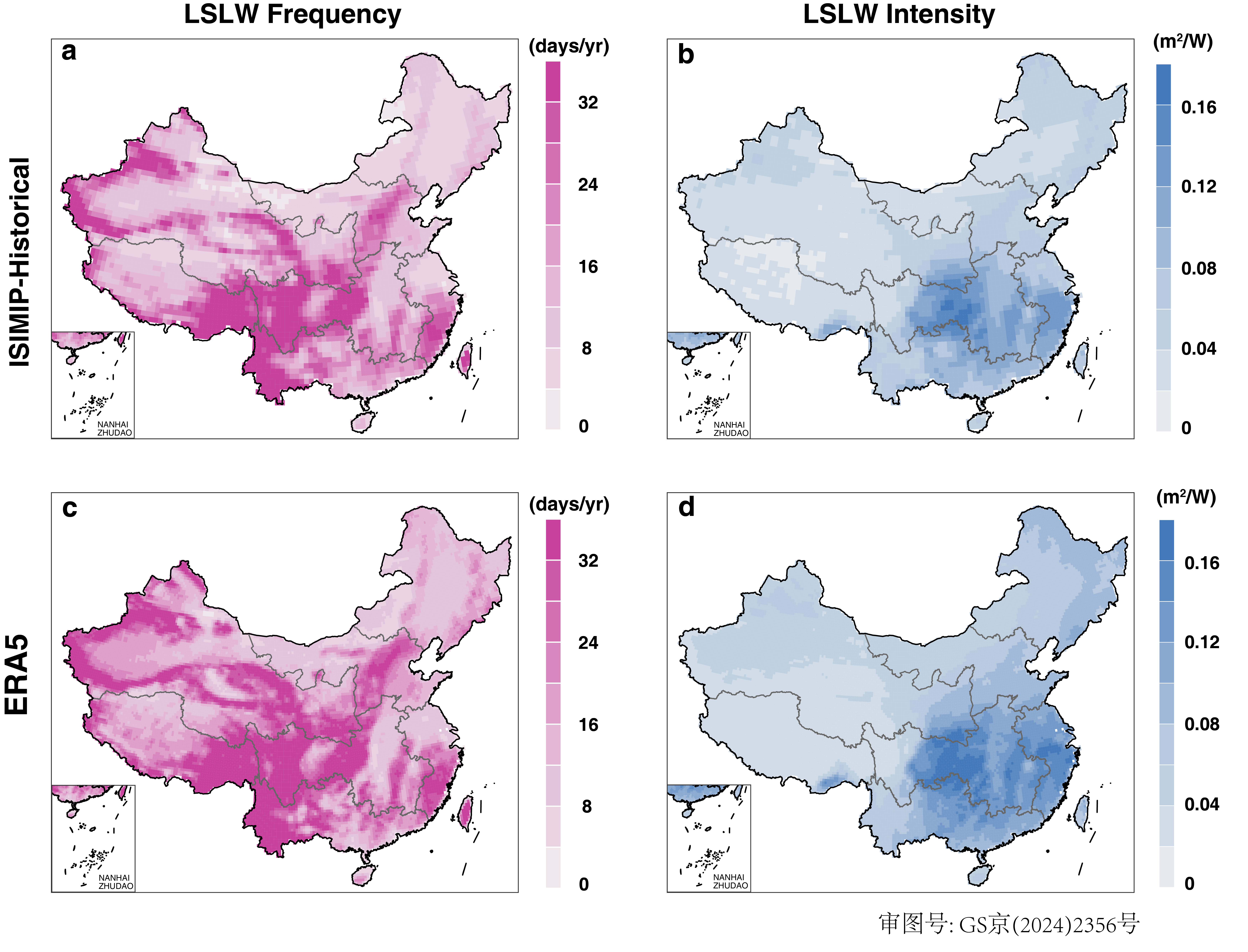


**Supplementary Figure S2.** The spatial distribution of compound low-solar-low-wind extremes’ (a, c) frequency (days/year) and (b, d) intensity (m^2^/W) over the (a, b) historical period (1961-1990) based on multi-model ensemble mean, and over the (c, d) same period based on the ERA5 reanalysis dataset. Black lines represent China’s seven regional power grids.


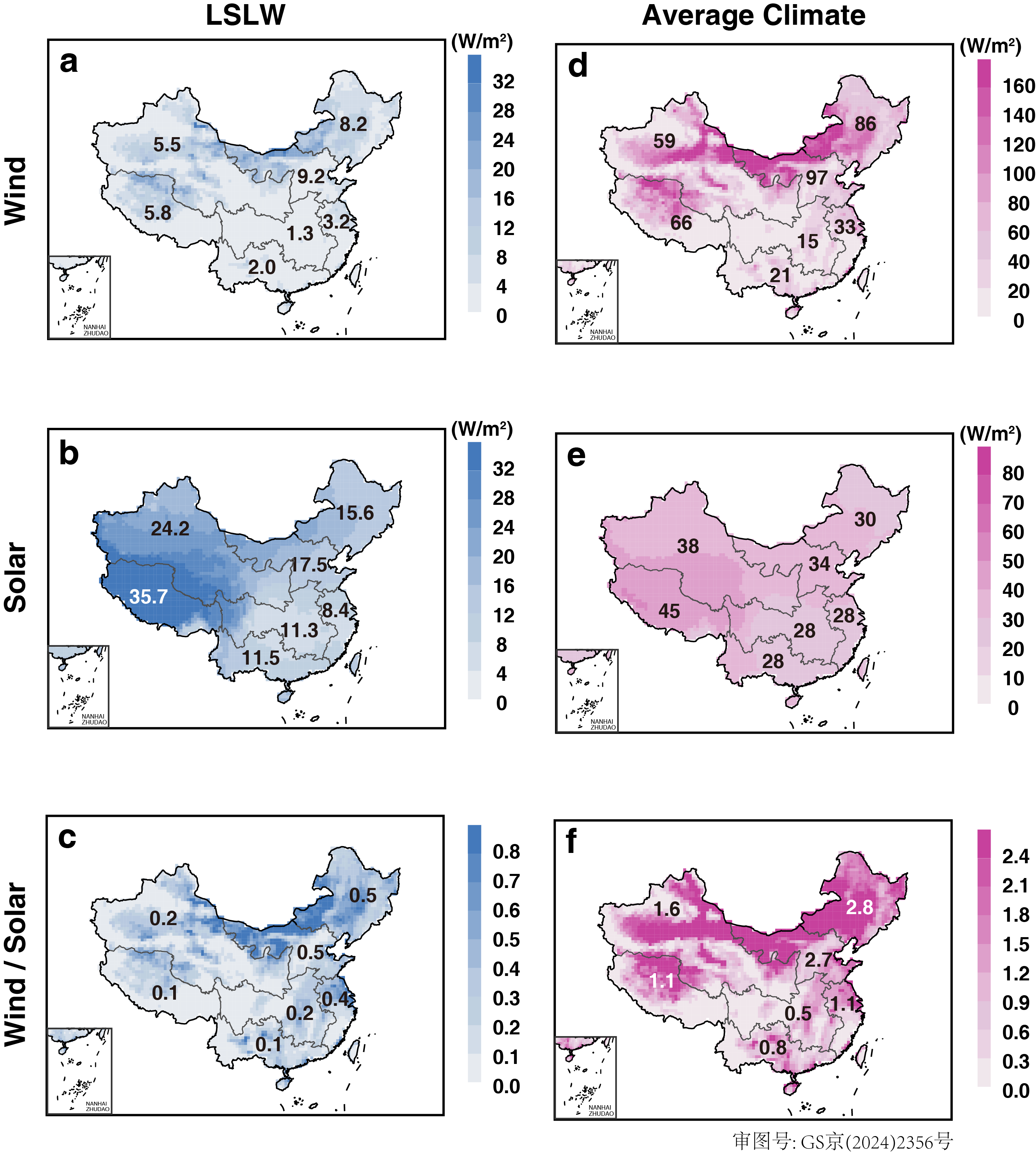


**Supplementary Figure S3.** The spatial distribution of (a, d) wind and (b, e) solar resource, and (c, f) their relative importance during (a-c) compound low-solar-low-wind (LSLW) extremes and under (d-f) average climate over the historical period (1961-1990).


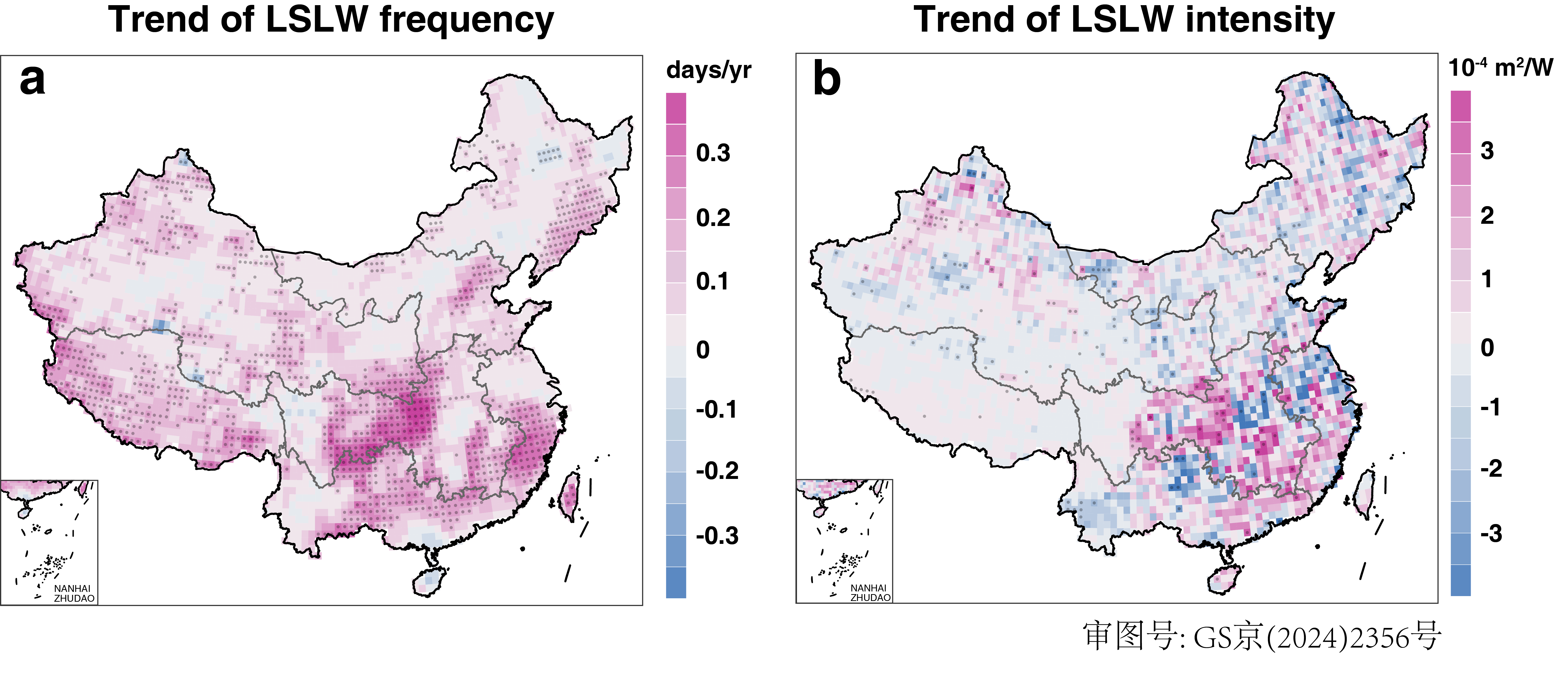


**Supplementary Figure S4.** The spatial distribution of trend in compound low-solar-low-wind (LSLW) extremes’ (a) frequency ((days/yr)/yr) and (b) intensity ((10^-4^ m^2^/W)/yr) during the historical period (1961-1990). The dots indicate the significant trends with p < 0.05 based on the Mann-Kendall test.


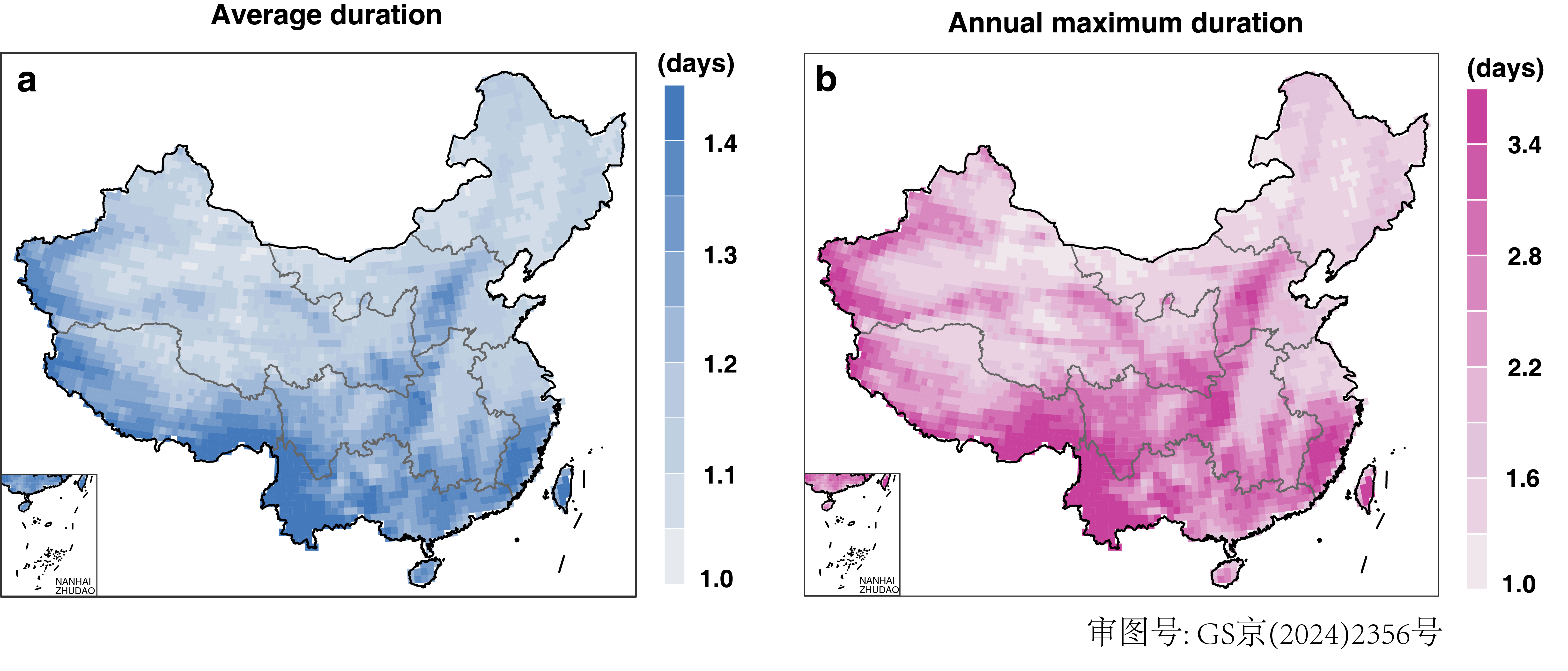


**Supplementary Figure S5.** The spatial distribution of compound low-solar-low-wind (LSLW) extremes’ (a) average duration (days) and (b) annual maximum duration (days) during the historical period (1961-1990).


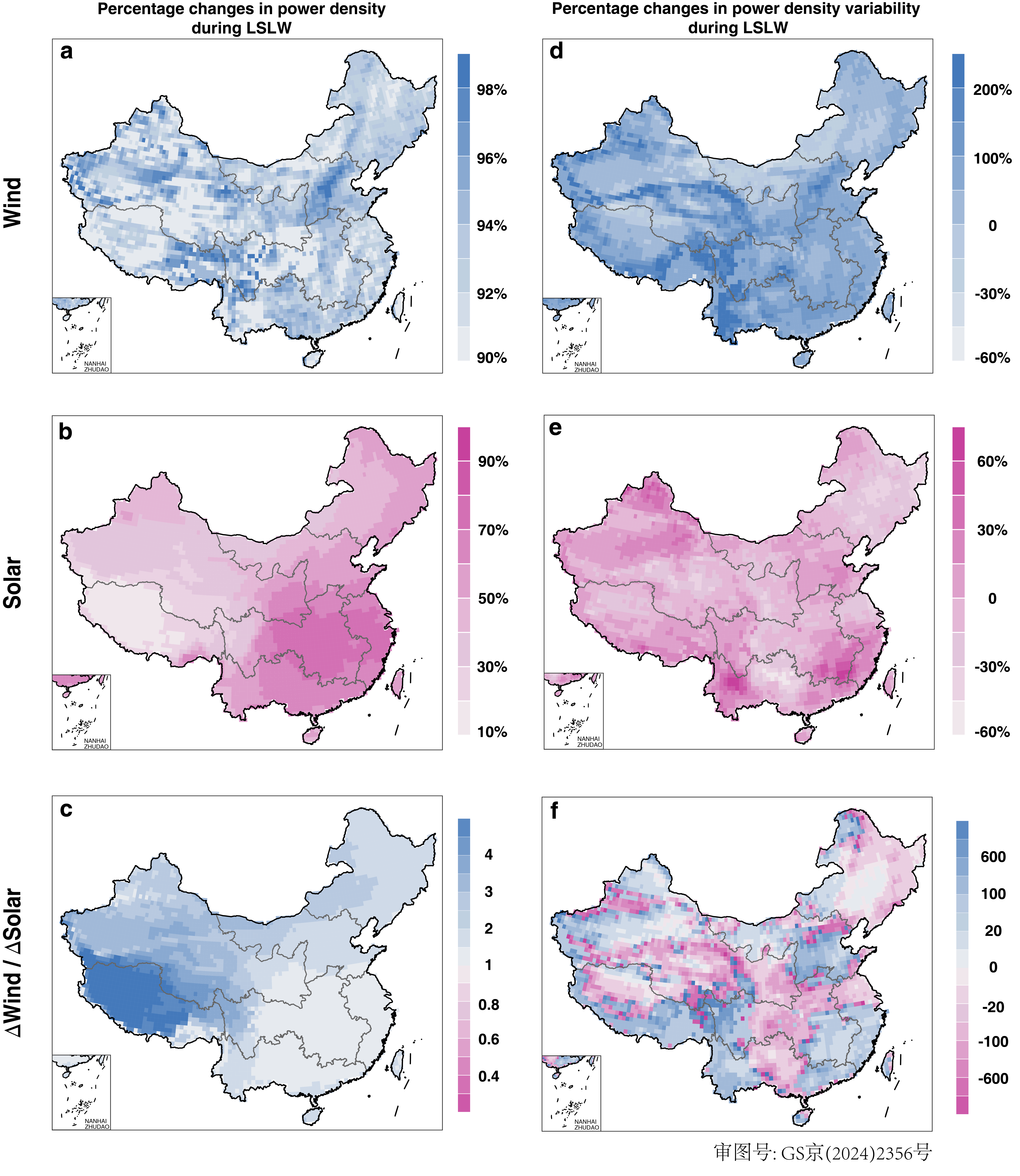


**Supplementary Figure S6.** Wind and solar power density and variability percentage changes during historical compound low-solar-low-wind (LSLW) extremes (1961-1990). The spatial distribution of (a) wind resource percentage reductions ((WE_average climate_-WE_LSLW_)/WE_average climate_), (b) solar resource percentage reductions ((PV_average climate_-PV_LSLW_)/PV_average climate_), (d) wind resource variability percentage changes ((CV_WE LSLW_-CV_WE average climate_)/CV_WE average climate_), and (e) solar resource variability percentage changes ((CV_PV LSLW_-CV_PV average climate_)/CV_PV average climate_), as well as the relative importance between wind and solar in (c) resource percentage reductions (WE_percentage reduction_/PV_percentage reduction_) and (f) variability changes (CVWE_percentage changes_/CVPV_percentage changes_) during compound extremes relative to average climate.


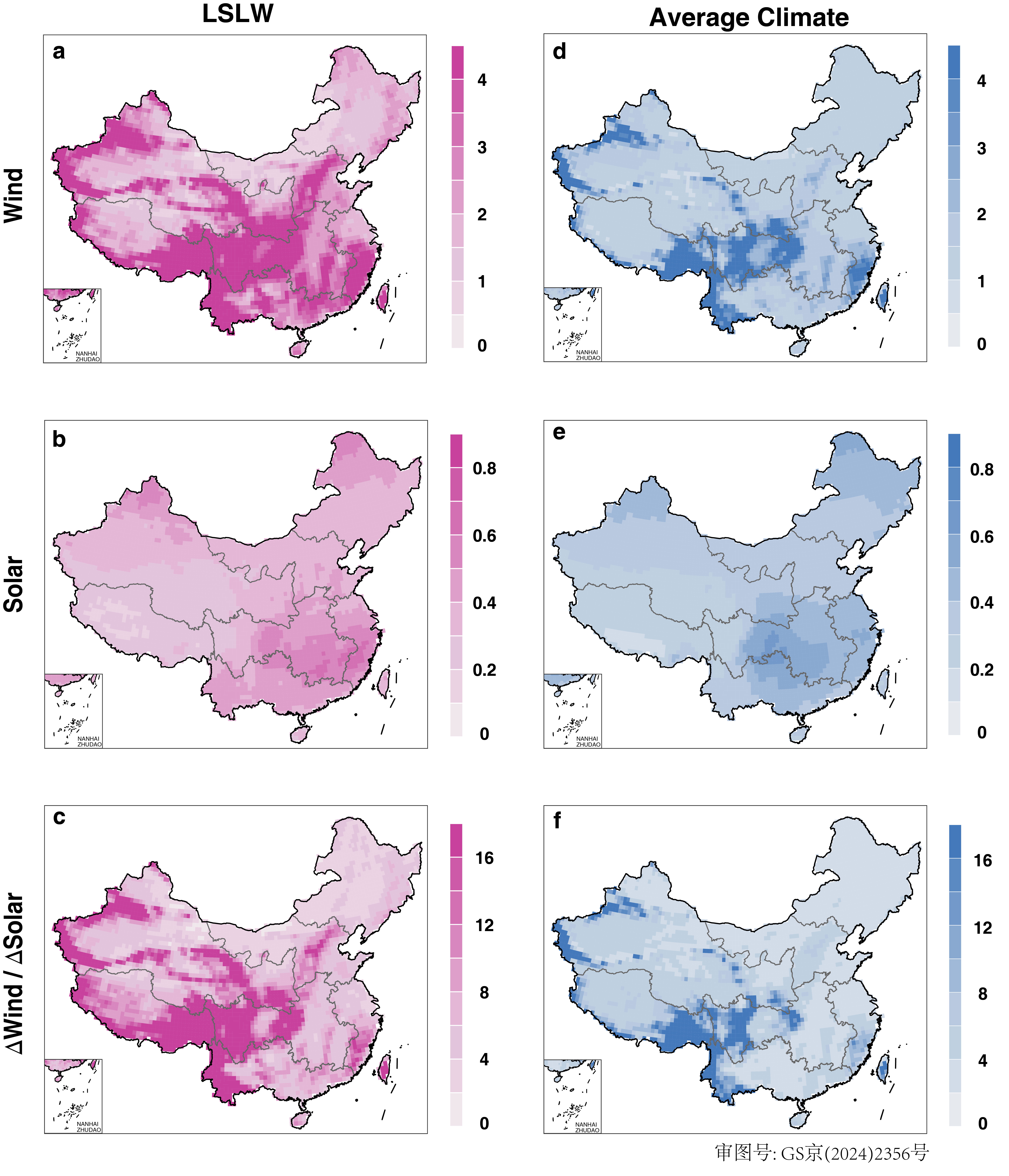


**Supplementary Figure S7**. The spatial distribution of (a, d) wind and (b, e) solar resource variability, and (c, f) their relative importance during (a-c) compound low-solar-low-wind (LSLW) extremes and under (d-f) average climate over the historical period (1961-1990).


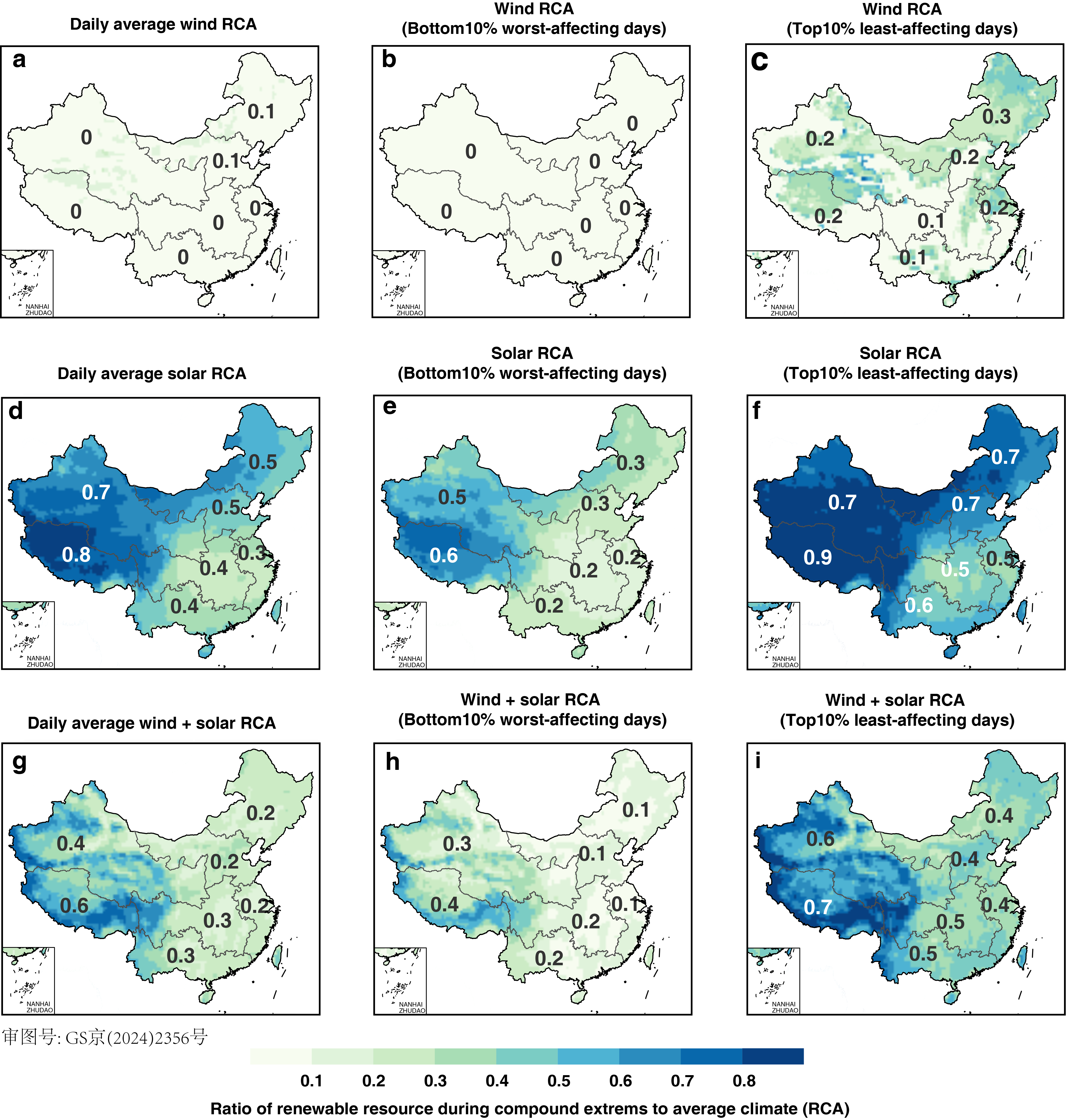


**Supplementary Figure S8**. Renewable energy resource deficiency during historical compound low-solar-low-wind extremes. The spatial distribution of the daily average ratio of the **R**emaining (a-c) wind, (d-f) solar, and (g-i) the sum of wind and solar energy resource during **C**ompound low-solar-low-wind extremes to their respective values under **A**verage climate for the period of 1961-1990 (RCA). RCA values for (a, d, g) daily mean, (b, e, h) the bottom 10% worst-affecting days with the lowest RCA, and (c, f, i) the top 10% least-affecting days with the highest RCA.


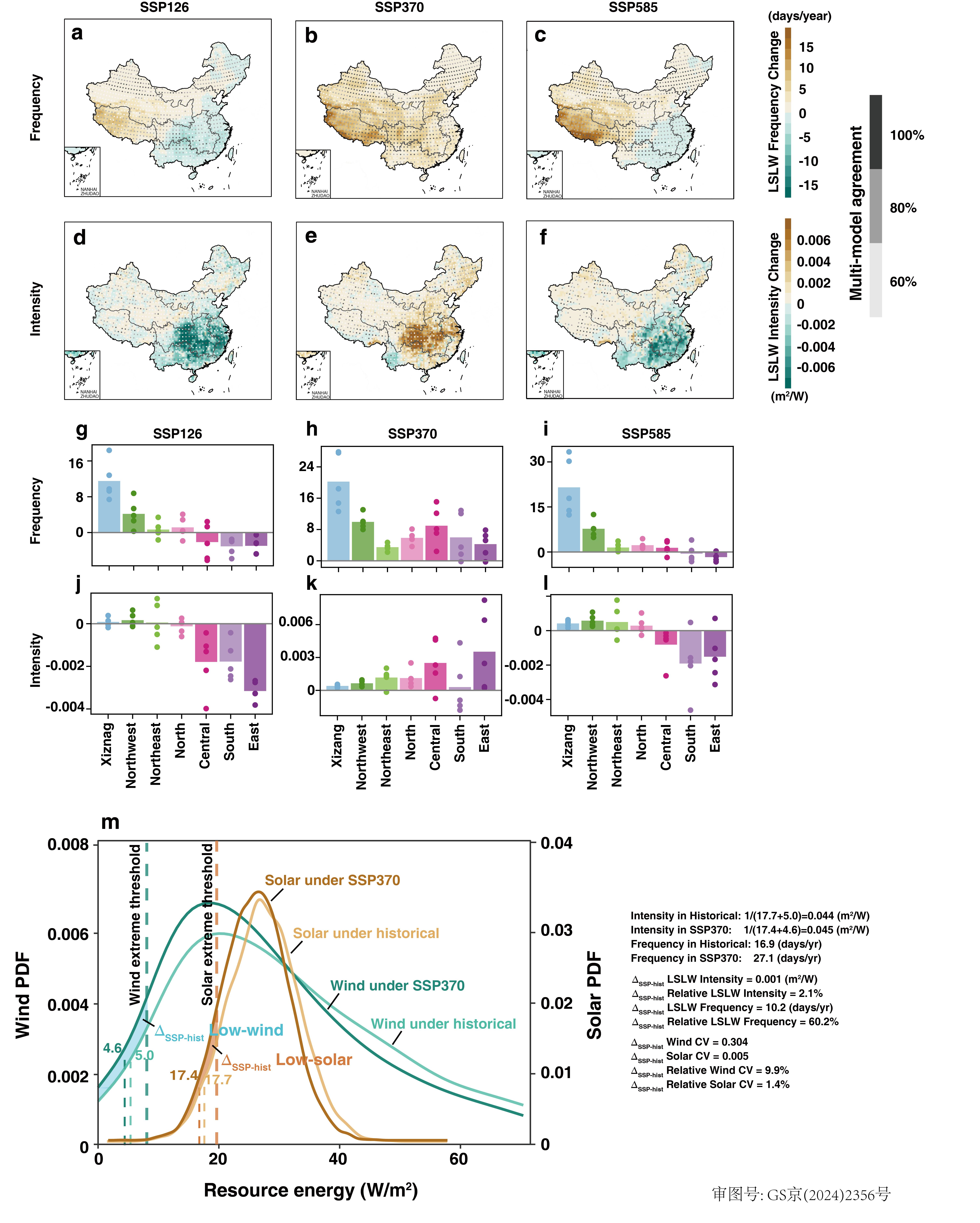


**Supplementary Figure S9**. Absolute changes in compound low-solar-low-wind (LSLW) extremes’ frequency (days/year) and intensity (m^2^/W) under climate change. Changes in the frequency (a-c) and intensity (d-f) of compound LSLW extremes under (a, d) SSP126, (b, e) SSP370, and (c, f) SSP585 scenarios over 2036-2065 relative to the historical period (1961-1990). Grey shading points denote exceeding 60% of models agree on the sign of change. Regional-average changes in the frequency (g-i, days/year) and intensity (j-l, m^2^/W) of compound LSLW extremes under (g, j) SSP126, (h, k) SSP370, and (i, l) SSP585 scenarios over 2036-2065 relative to the historical period (1961-1990). The points are individual model values and the bars represent the mean values of five climate models. (m) Schematic diagram of the probability distribution of wind (brown) and solar (green) resource averaged across the country under SSP370 scenario over 2036-2065 and historical scenario over 1961-1990. Wind and solar PDF refers to probability density function that describes the probability distribution for the wind (left and brown) and solar (right and green) resource.


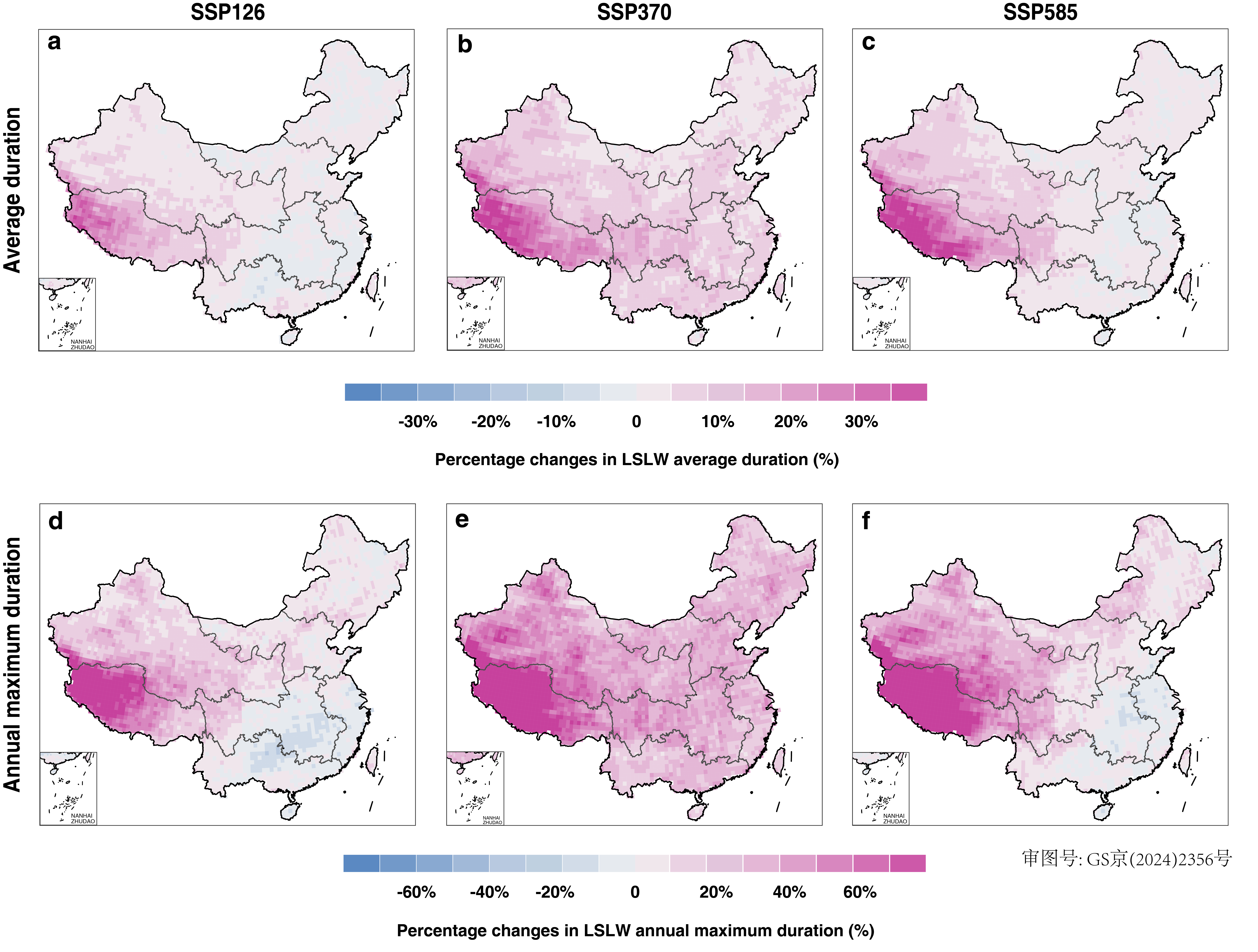


**Supplementary Figure S10**. Percentage changes (%) in compound low-solar-low-wind (LSLW) extremes’ (a-c) average duration and (d-f) annual maximum duration under (a, d) SSP126, (b, e) SSP370, and (c, f) SSP585 scenarios over 2036-2065 relative to the historical period (1961-1990).


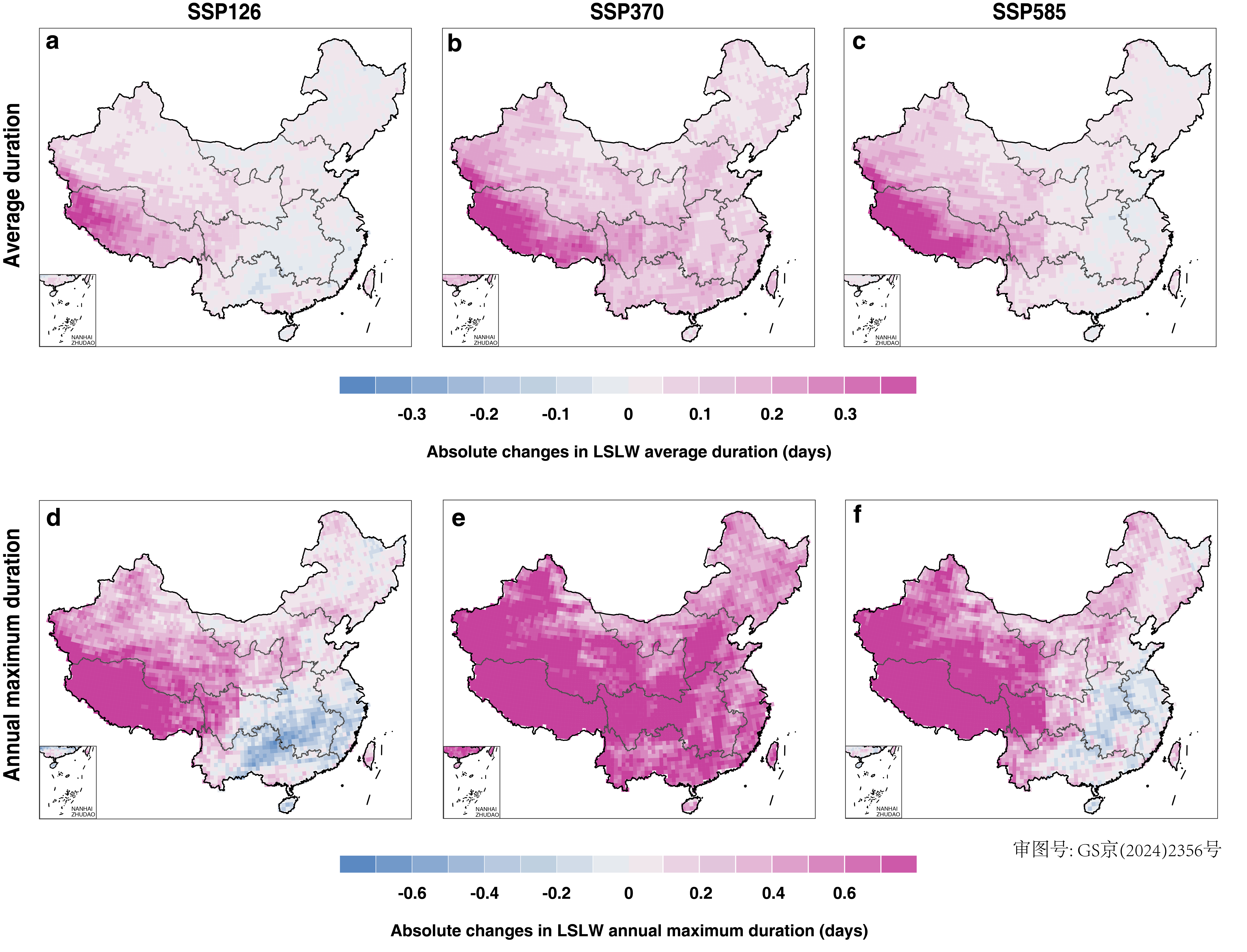


**Supplementary Figure S11**. Absolute changes in compound low-solar-low-wind (LSLW) extremes’ (a-c) average duration (days) and (d-f) annual maximum duration (days) under (a, d) SSP126, (b, e) SSP370, and (c, f) SSP585 scenarios over 2036-2065 relative to the historical period (1961-1990).


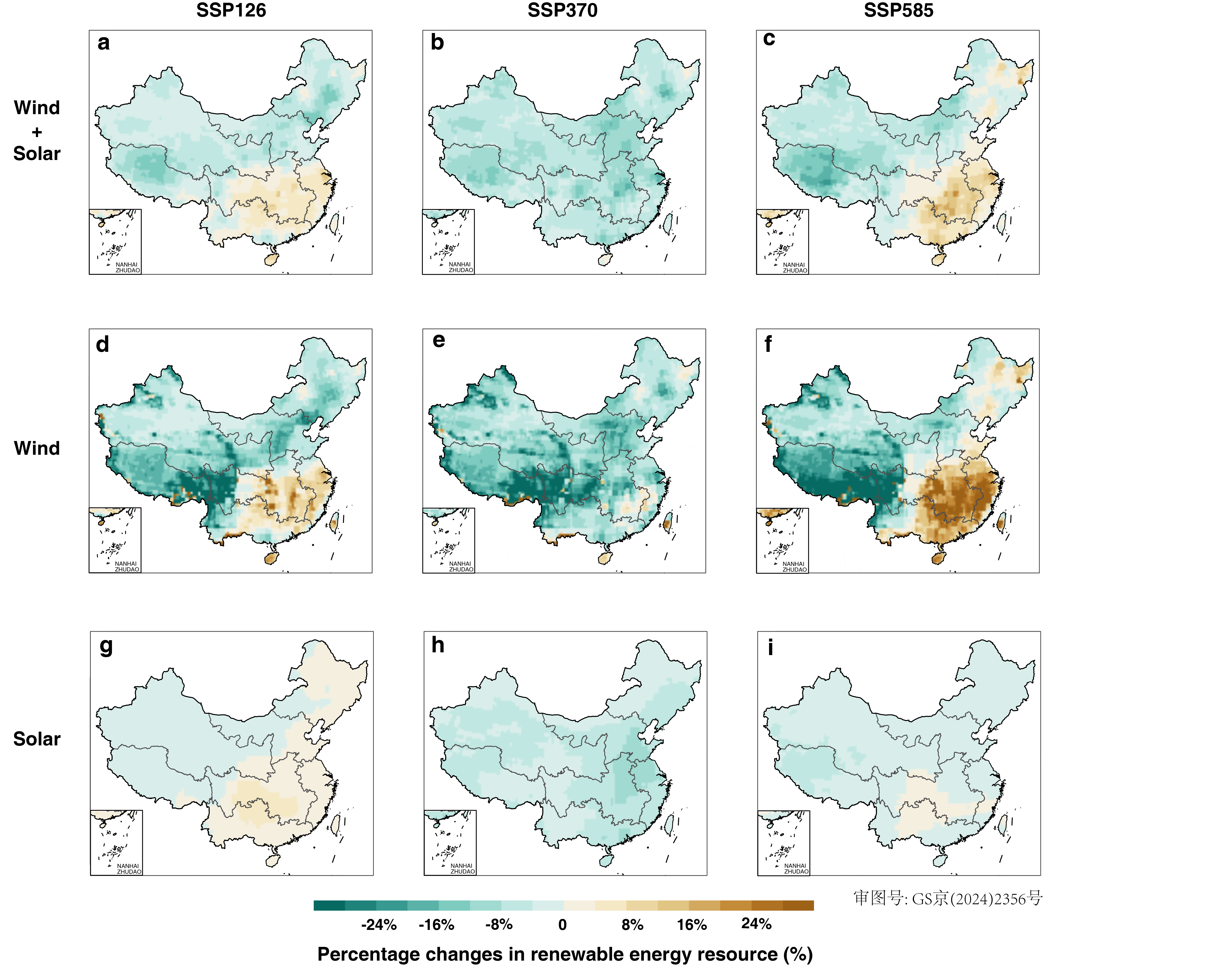


**Supplementary Figure S12**. Percentage changes (%) (a-c) in the sum of wind and solar, (d-f) wind, and (g-h) solar energy resource under (a, d, g) SSP126, (b, e, h) SSP370, and (c, f, i) SSP585 scenarios over 2036-2065 relative to the historical period (1961-1990).


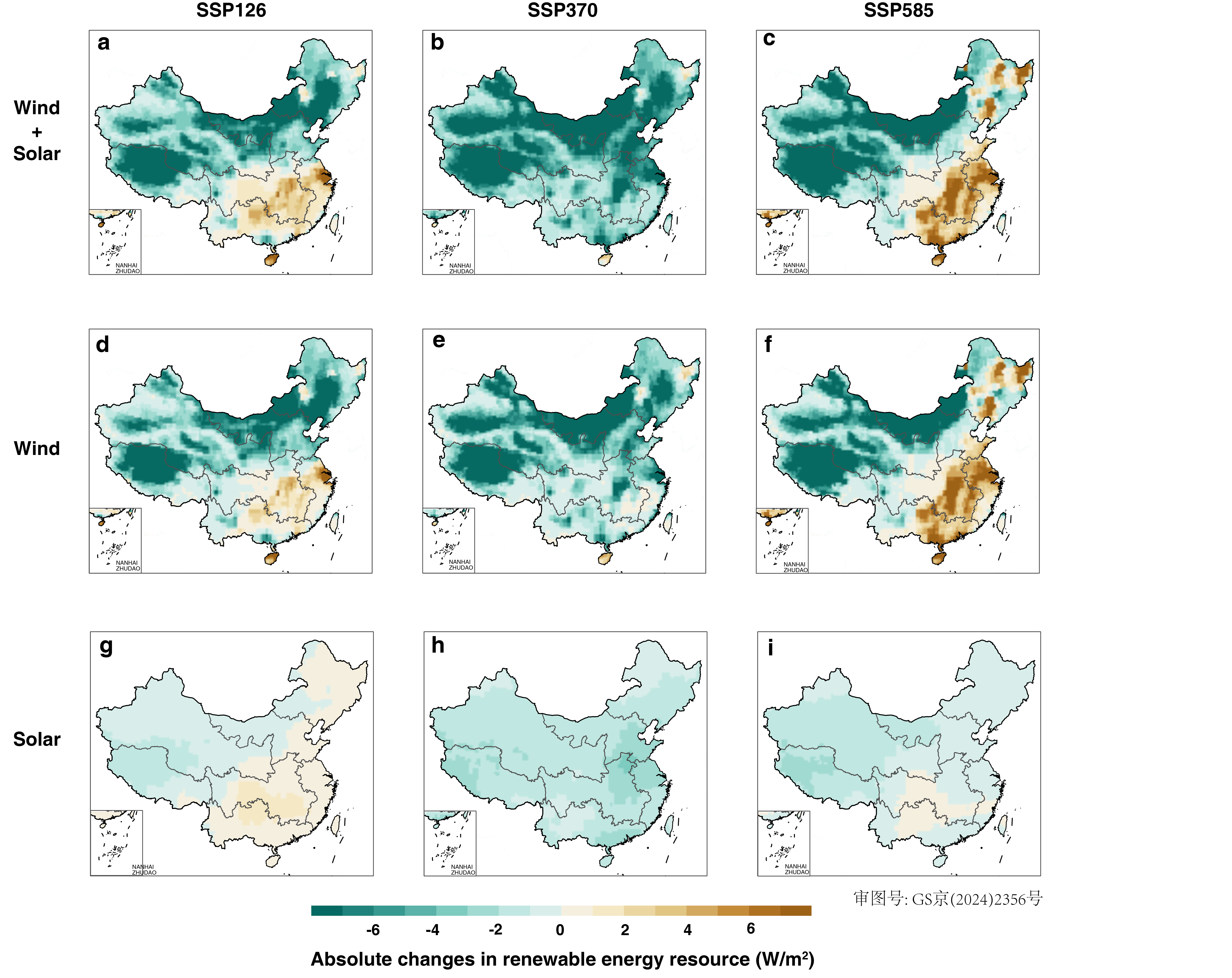


**Supplementary Figure S13**. Absolute changes (a-c) in the sum of wind and solar (W/m^2^), (d-f) wind (W/m^2^) and (g-h) solar energy resource (W/m^2^) under (a, d, g) SSP126, (b, e, h) SSP370, and (c, f, i) SSP585 scenarios over 2036-2065 relative to the historical period (1961-1990).


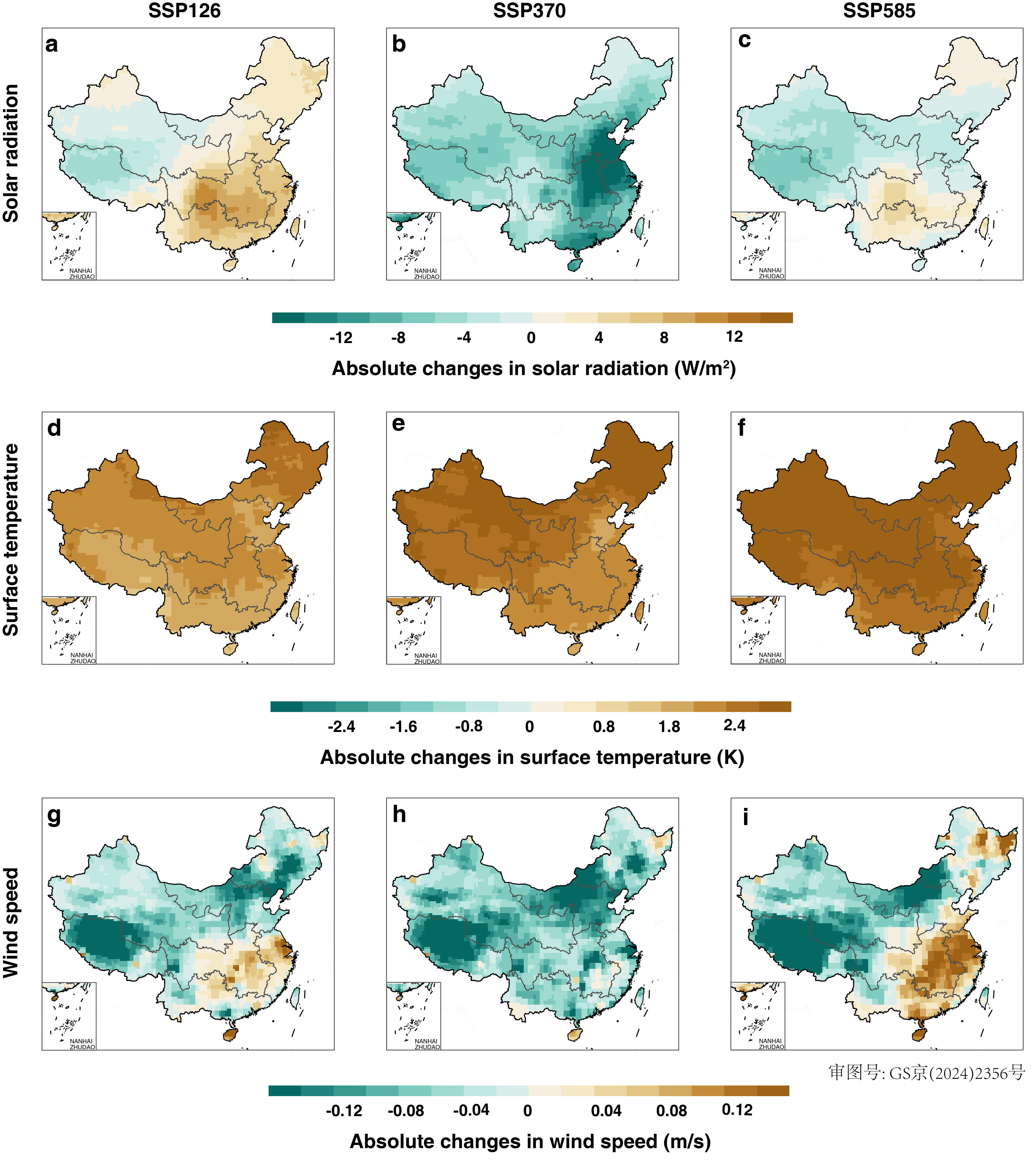


**Supplementary Figure S14**. Absolute changes in (a-c) solar radiation (W/m^2^), (d-f) surface temperature (K), and (g-i) wind speed (m/s) under (a, d, g) SSP126, (b, e, h) SSP370, and (c, f, i) SSP585 scenarios over 2036-2065 relative to the historical period (1961-1990).


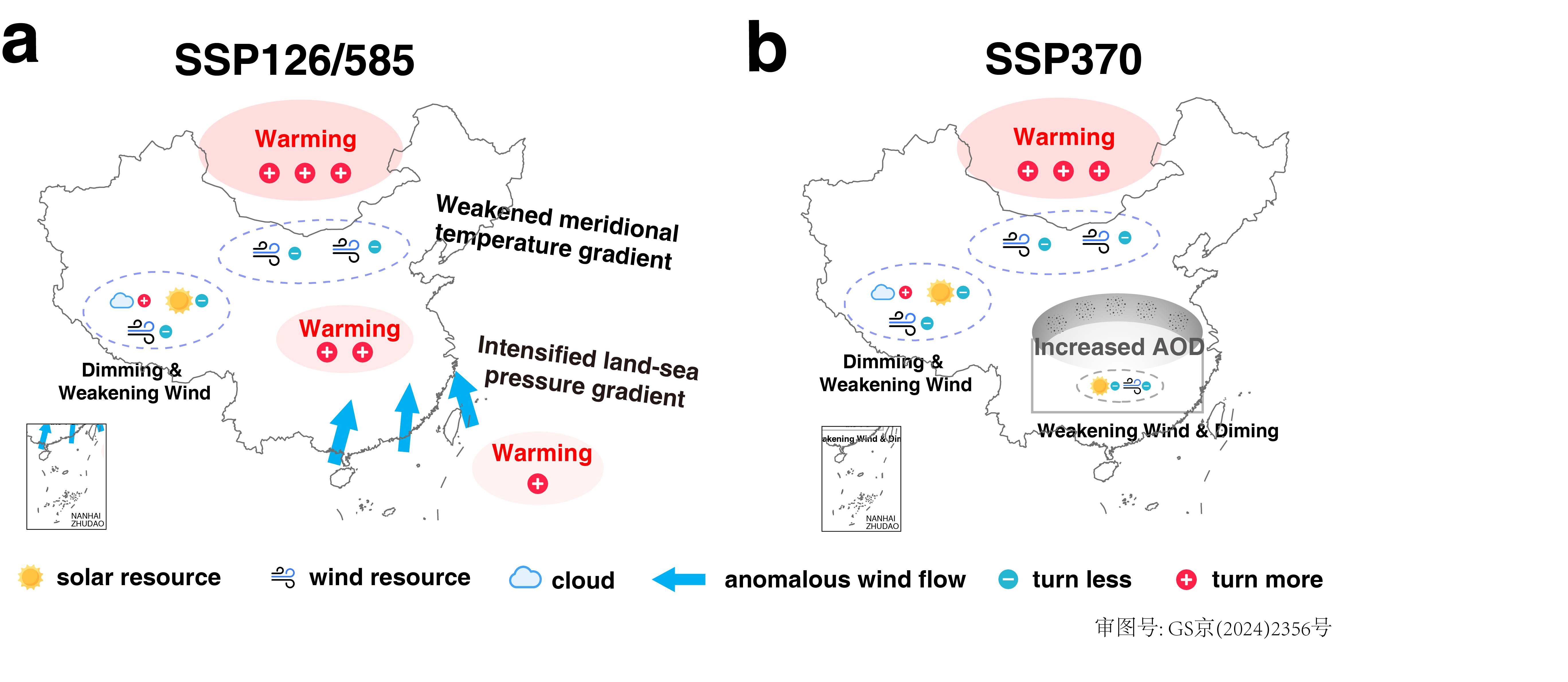


**Supplementary Figure S15.** Schematic diagram of the dynamic mechanism for changes in wind speed and solar irradiance under (a) SSP126/585 and (b) SSP370. Over mid-latitude northern China across all scenarios, future warming weakens the driving force (i.e., pressure-gradient force) by diminishing the meridional temperature gradient, and ultimately reduce surface wind speed. The increased frequency of extremely low surface solar radiation aligns with the overall decline in surface solar radiation, primarily driven by the increased frequency of extremely high total cloud cover. Over southern China, increasing wind speed and solar radiation lead to a slight decline in the frequency of LSLW under SSP126 and SSP370, which respectively, arises from the enhanced East Asian summer monsoon (as indicated by the intensified land-sea pressure gradient) and the decrease in dense cloud cover. Conversely in SSP370, the distinctively high aerosol loading dominantly shapes the dimming and weakening of surface wind.


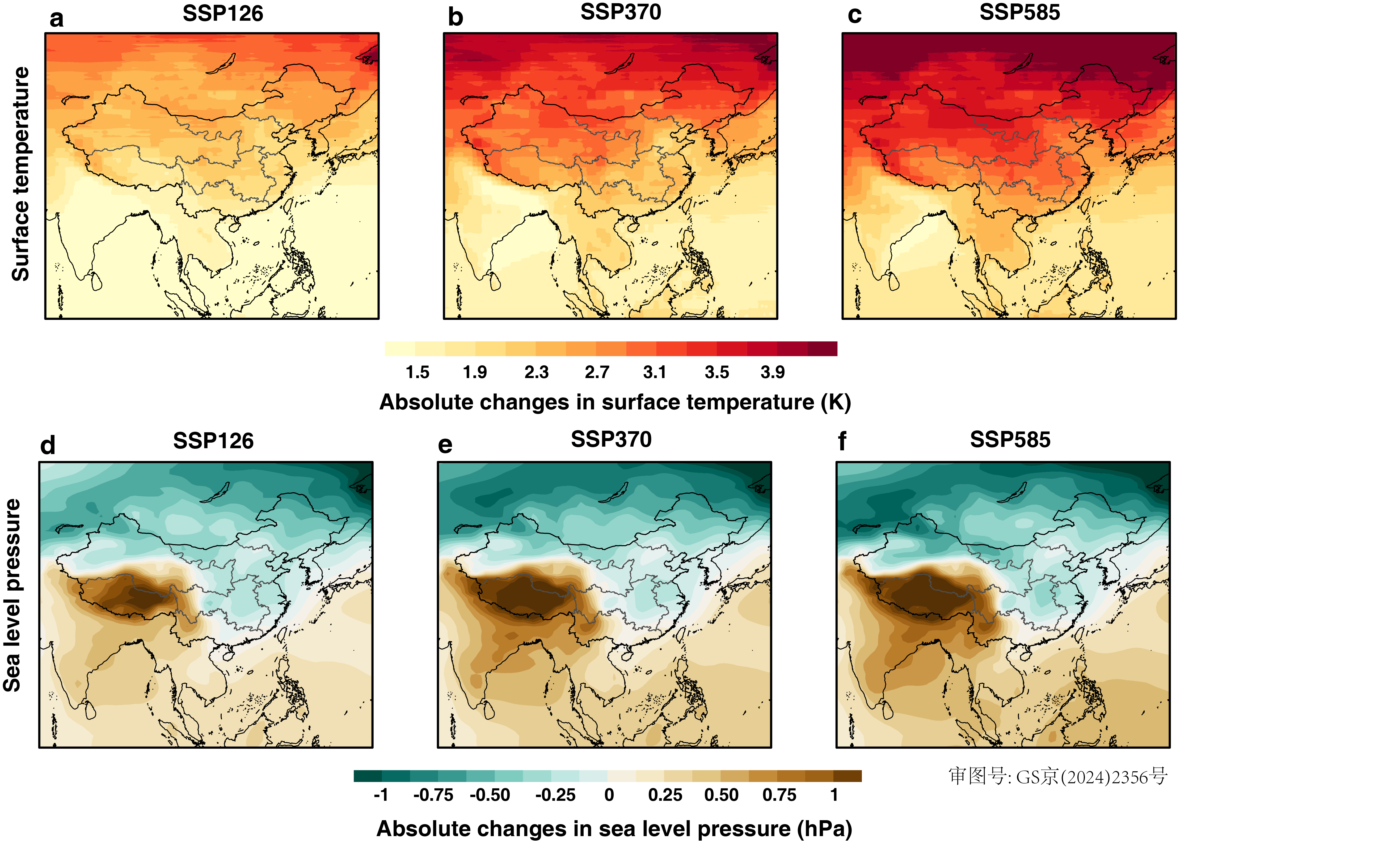


**Supplementary Figure S16**. Absolute changes in (a-c) surface temperature (K), (d-f) sea level pressure (hPa) under (a, d) SSP126, (b, e) SSP370, and (c, f) SSP585 scenarios over 2036-2065 relative to the historical period (1961-1990).


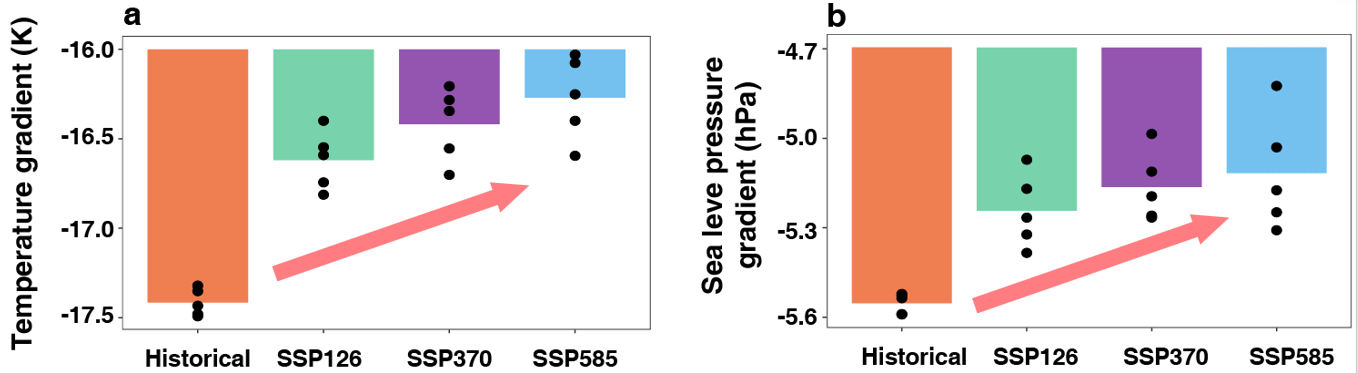


**Supplementary Figure S17**. The meridional (a) surface temperature (K) and (b) sea level pressure gradients (hPa) between low and mid latitudes in East Asia under historical, SSP126, SSP370, and SSP585 scenarios. We define 2 latitude zones: LZ1 (15-30°N, 70-135°E) and LZ2 (30-50°N, 70-135°E). The surface temperature (pressure) gradient is calculated as the difference between the surface temperature (pressure) between LZ2 and LZ1. The points are individual model values and the bars represent the mean values of five climate models.


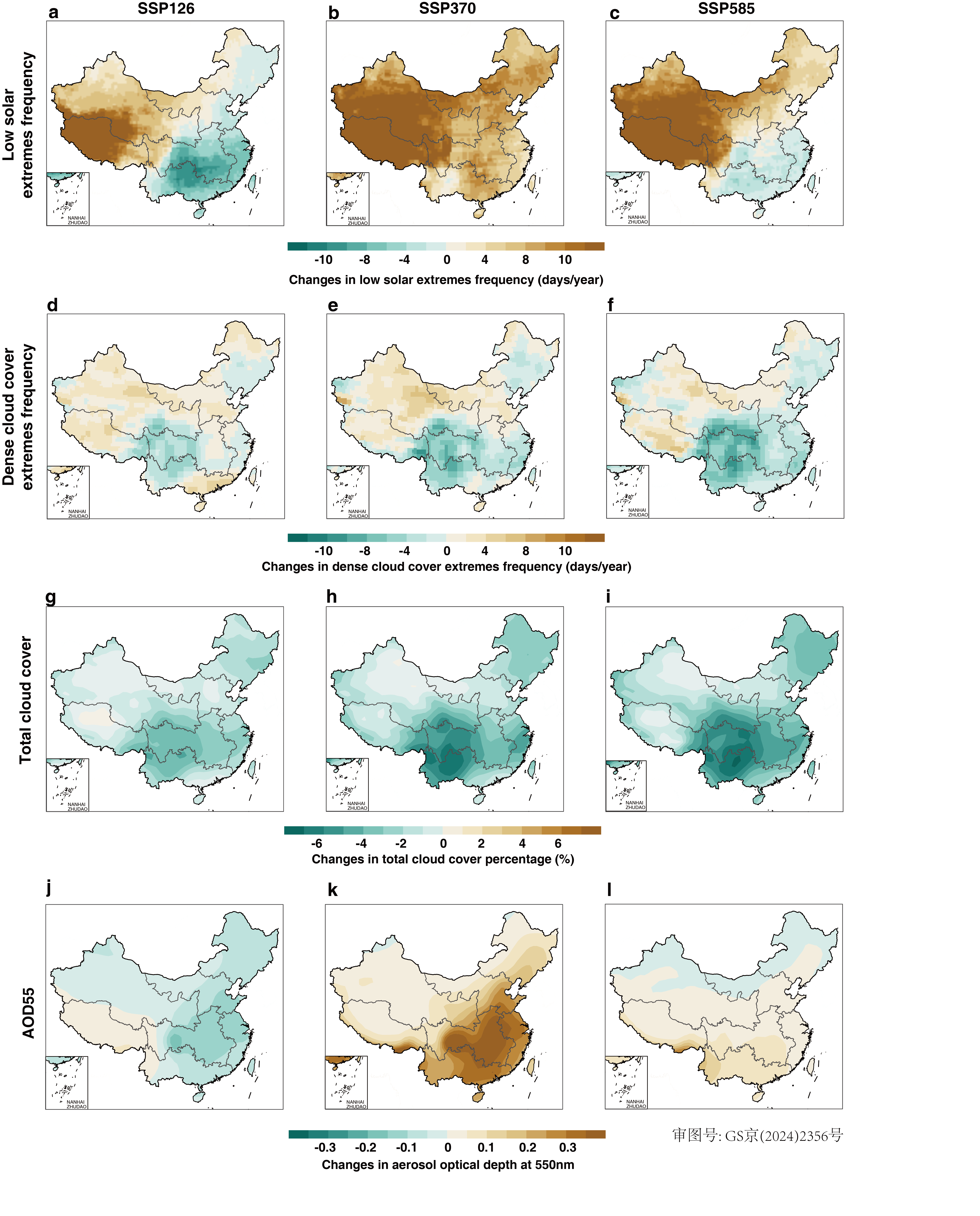


**Supplementary Figure S18**. Changes in (a-c) low solar extremes frequency (days/year), (d-f) dense total cloud cover extremes frequency (days/year), (g-i) total cloud cover percentage (%), (j-l) aerosol optical depth (AOD) at 550 nm under (a, d, g, i) SSP126, (b, e, h, k) SSP370, and (c, f, i, l) SSP585 scenarios over 2036-2065 relative to the historical period (1961-1990). Dense total cloud cover is defined as exceeding the 90^th^ percentile threshold of total cloud cover.


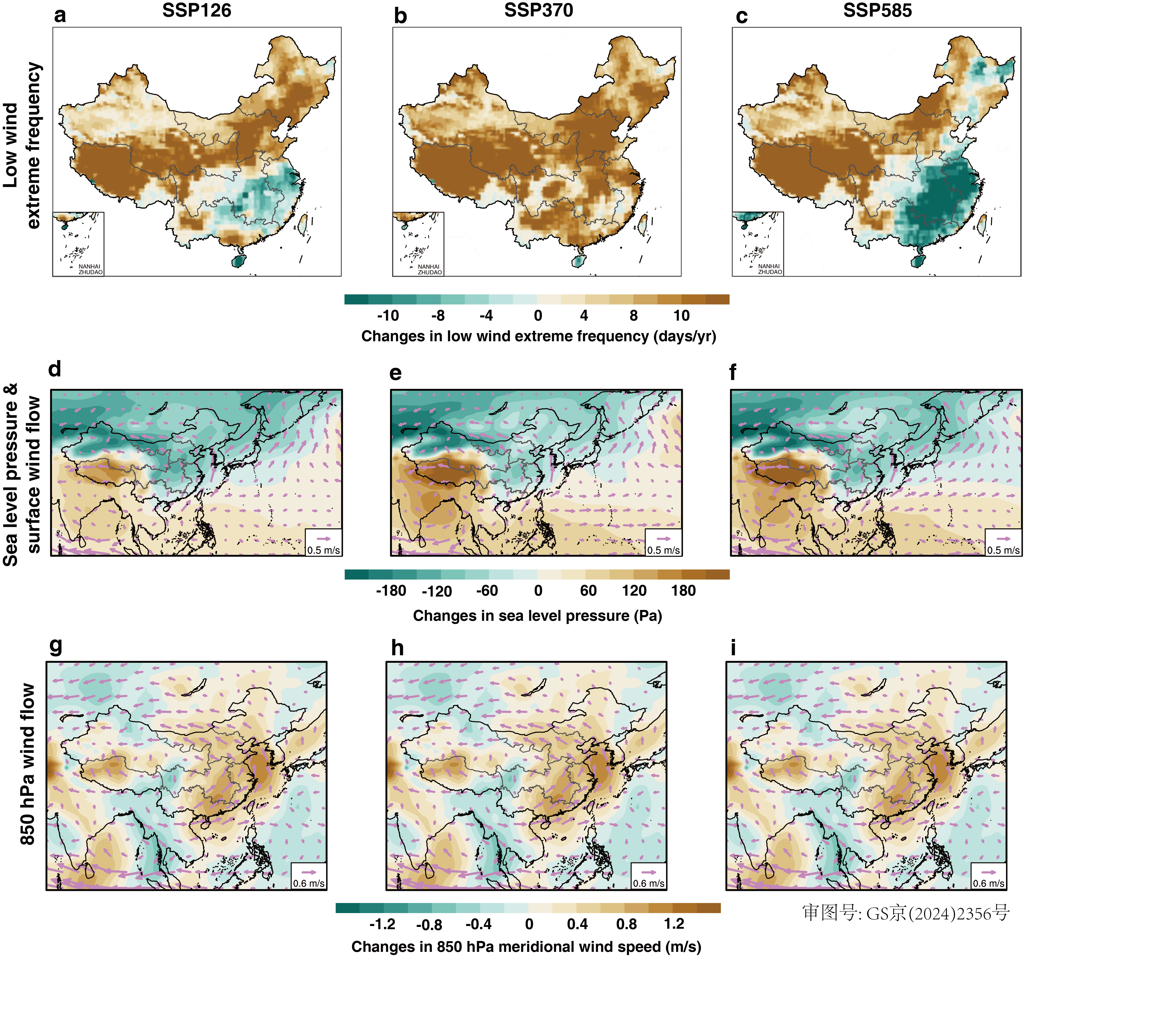


**Supplementary Figure S19**. Changes in (a-c) low wind extremes frequency (days/year), (d-f) sea level pressure (Pa, shading) and surface wind flow (m/s, vector) in June-July-August (JJA), (g-i) meridional wind speed (m/s, shading) and wind flow anomalies at 850 hPa in JJA under (a, d, g) SSP126, (b, e, h) SSP370, and (c, f, i) SSP585 scenarios over 2036-2065 relative to the historical period (1961-1990).


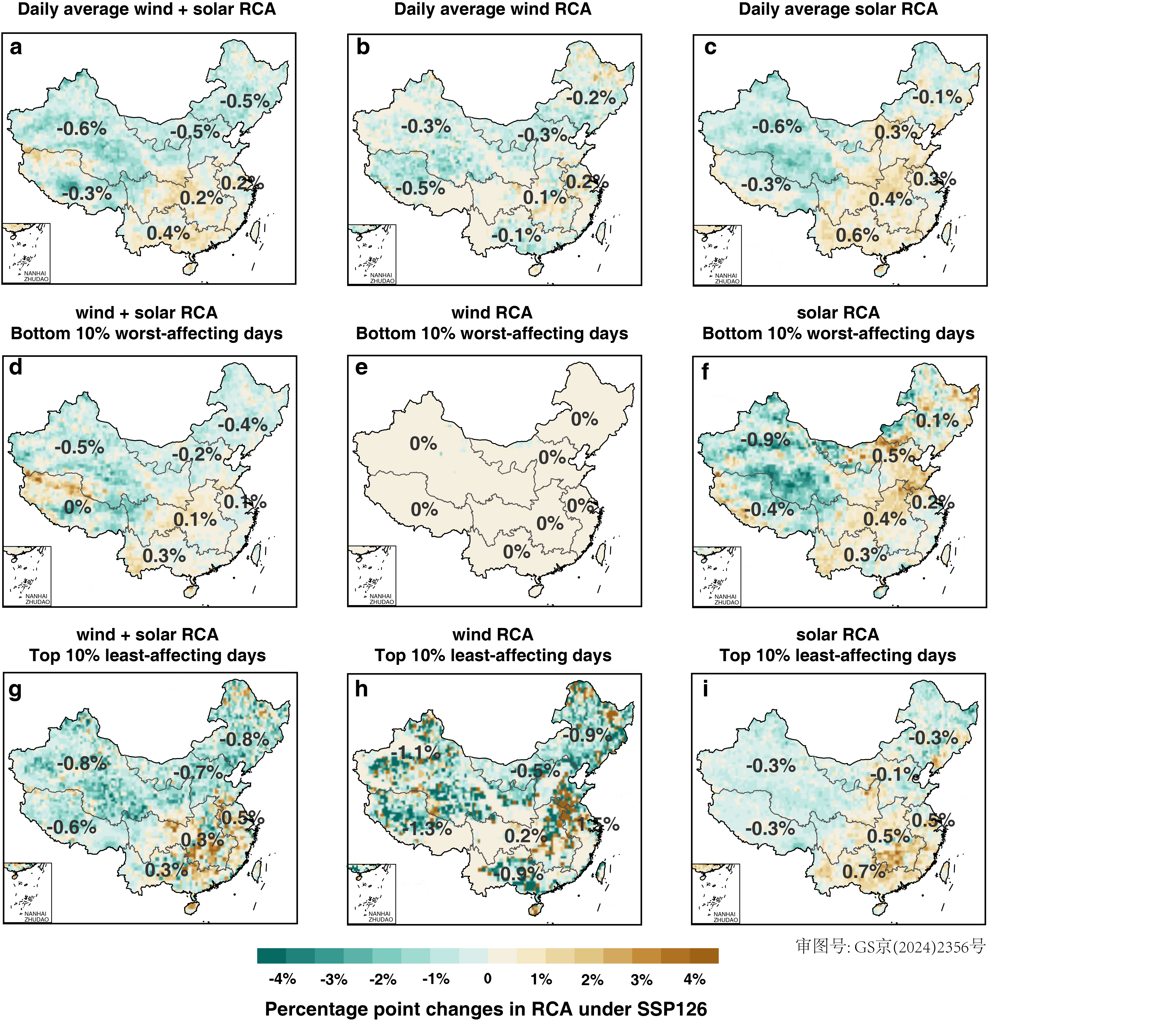


**Supplementary Figure S20**. Percentage point changes in RCA under SSP126 scenario over 2036-2065 relative to the historical period (1961-1990). RCA values for (a-c) daily mean, (d-f) the bottom 10% worst-affecting days with the lowest RCA, and (g-i) the top 10% least-affecting days with the highest RCA of the (a, d, g) sum of wind and solar energy resource, (b, e, h) wind, and (c, f, i) solar, repectively. RCA refers to the ratio of **R**emaining energy resource during **C**ompound low-solar-low-wind extrems under SSP126 scenario over 2036-2065 to that under **A**verage climate for the period of 1961-1990.


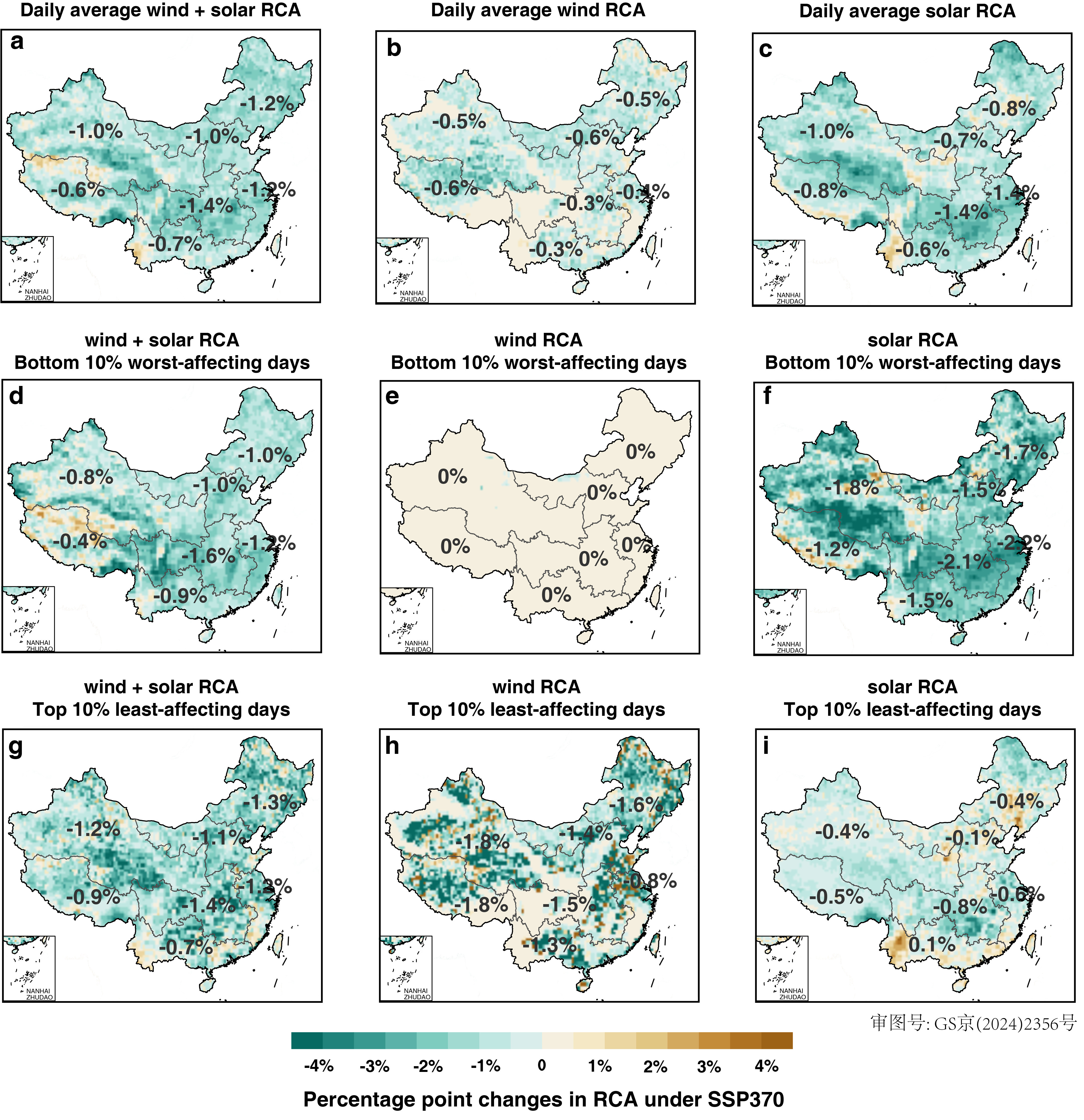


**Supplementary Figure S21**. Percentage point changes in RCA under SSP370 scenario over 2036-2065 relative to the historical period (1961-1990). RCA values for (a-c) daily mean, (d-f) the bottom 10% worst-affecting days with the lowest RCA, and (g-i) the top 10% least-affecting days with the highest RCA of the (a, d, g) sum of wind and solar energy resource, (b, e, h) wind, and (c, f, i) solar, repectively. RCA refers to the ratio of **R**emaining energy resource during **C**ompound low-solar-low-wind extrems under SSP370 scenario over 2036-2065 to that under **A**verage climate for the period of 1961-1990.


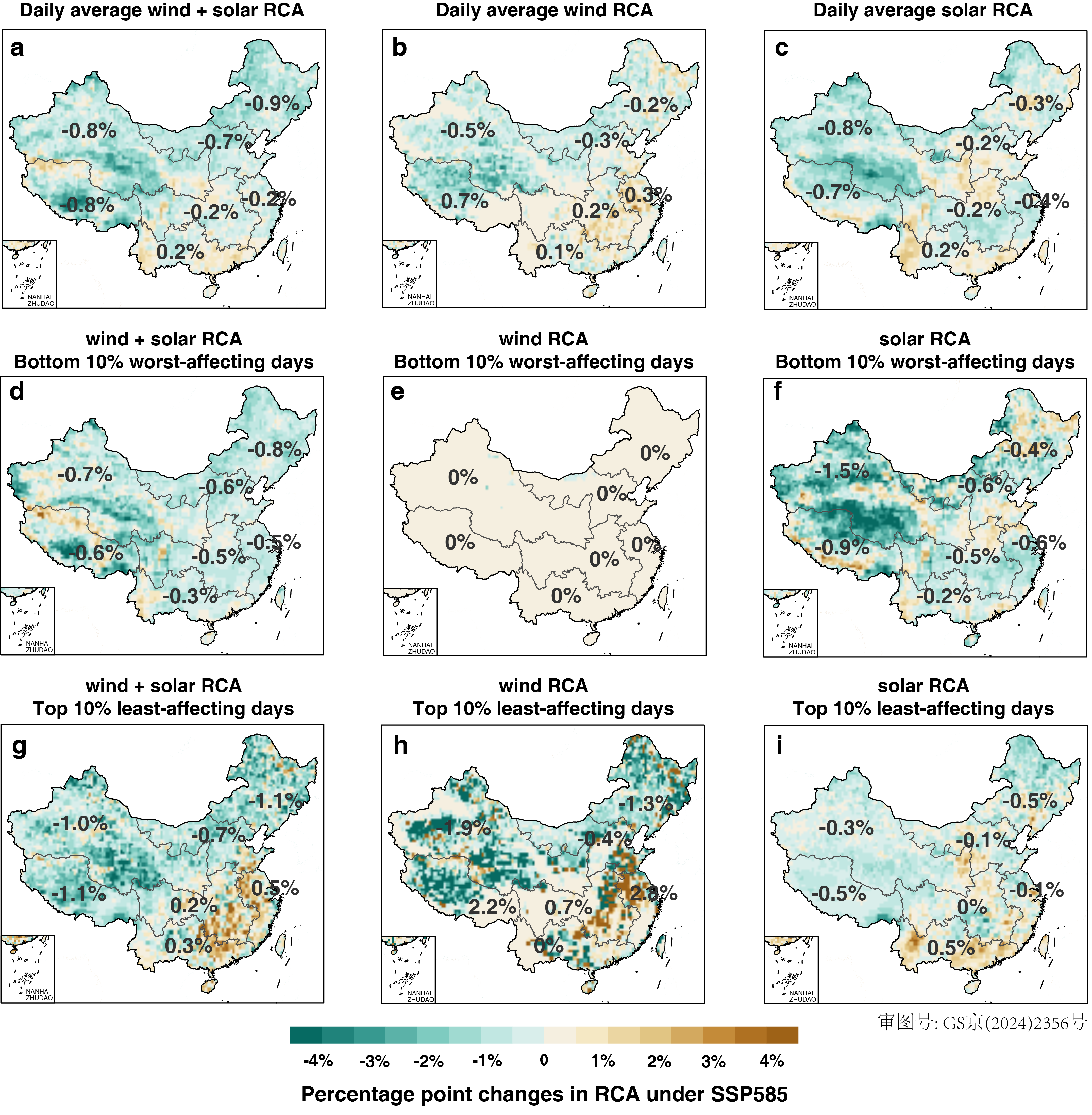


**Supplementary Figure S22**. Percentage point changes in RCA under SSP585 scenario over 2036-2065 relative to the historical period (1961-1990). RCA values for (a-c) daily mean, (d-f) the bottom 10% worst-affecting days with the lowest RCA, and (g-i) the top 10% least-affecting days with the highest RCA of the (a, d, g) sum of wind and solar energy resource, (b, e, h) wind, and (c, f, i) solar, repectively. RCA refers to the ratio of **R**emaining energy resource during **C**ompound low-solar-low-wind extrems under SSP585 scenario over 2036-2065 to that under **A**verage climate for the period of 1961-1990.


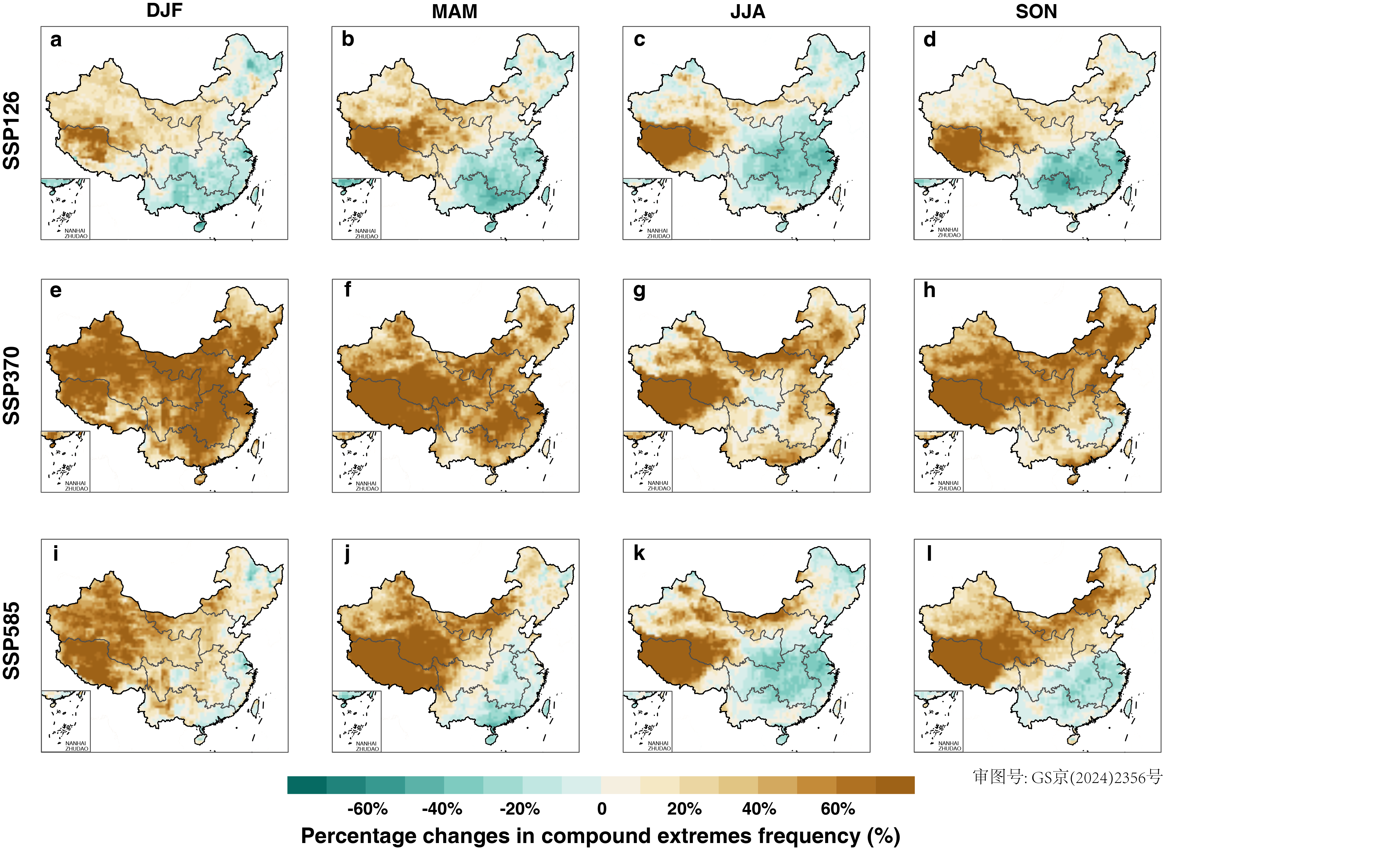


**Supplementary Figure S23**. Percentage changes (%) in the frequency of compound low-solar-low-wind extremes under (a-d) SSP126, (e-h) SSP370, and (i-l) SSP585 scenarios over 2036-2065 relative to the historical period (1961-1990) for each season: December-January-February (DJF, a, e, i); March-Apri-May (MAM, b, f, j); June-July-August (JJA, c, g, k); and September-October-November (SON, d, h, l).


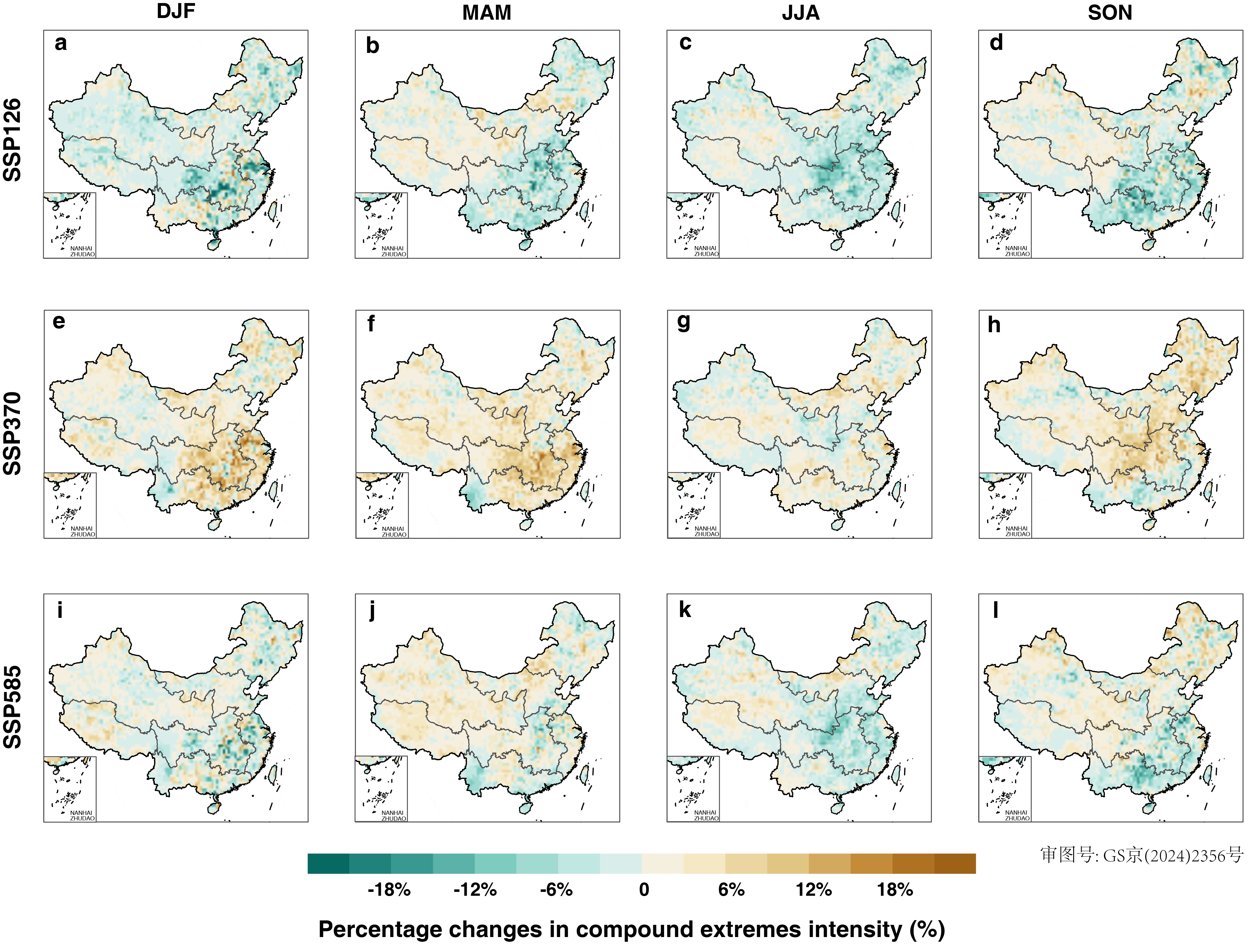


**Supplementary Figure S24**. Percentage changes (%) in the intensity of compound low-solar-low-wind extremes under (a-d) SSP126, (e-h) SSP370, and (i-l) SSP585 scenarios over 2036-2065 relative to the historical period (1961-1990) for each season: December-January-February (DJF, a, e, i); March-Apri-May (MAM, b, f, j); June-July-August (JJA, c, g, k); and September-October-November (SON, d, h, l).


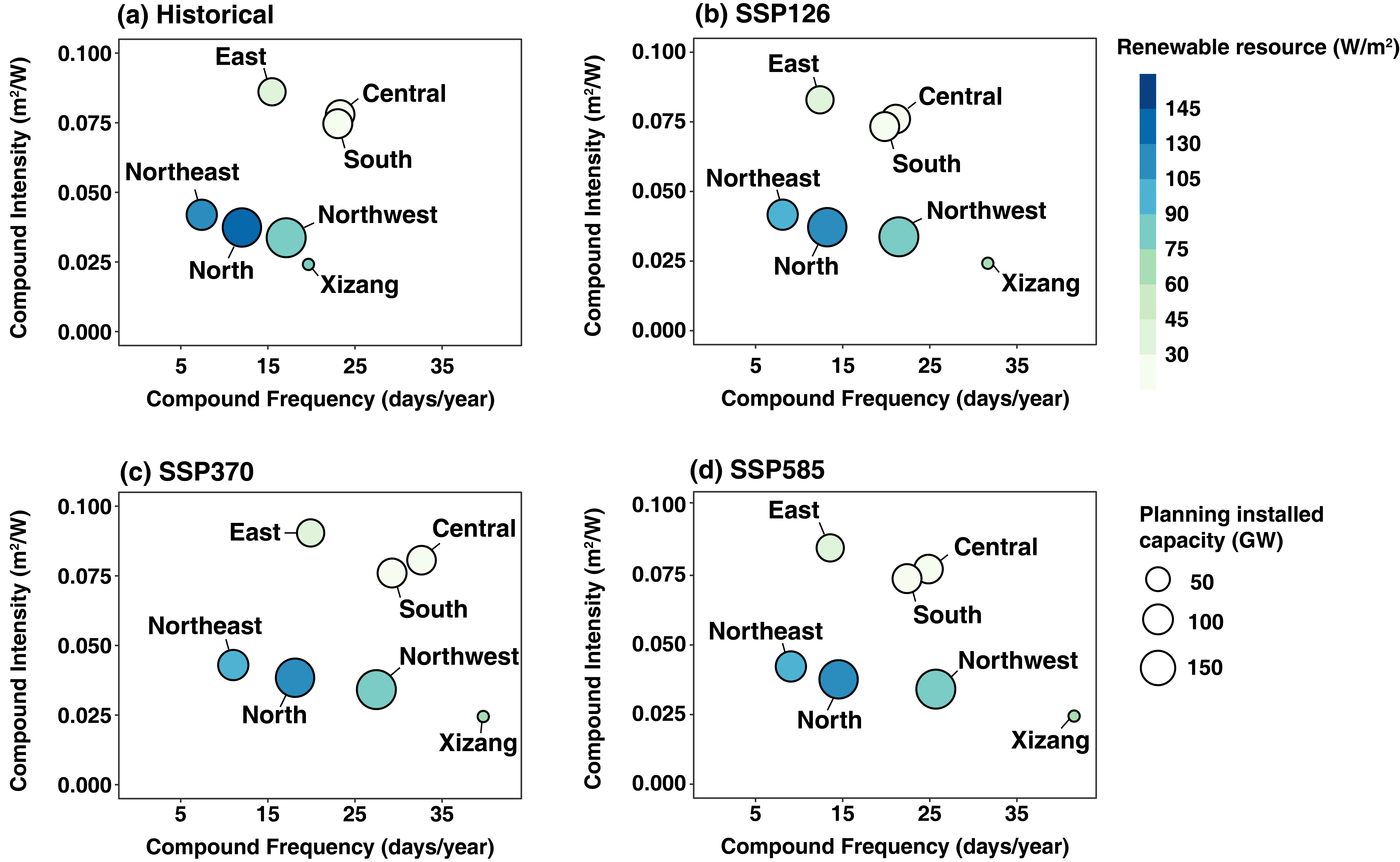


**Supplementary Figure S25**. Regional average of compound low-solar-low-wind extremes’ frequency (x-axis, days/year), compound low-solar-low-wind extremes’ intensity (y-axis, m^2^/W), and renewable resource (colors) under (a) Historical, (b) SSP126, (c) SSP370, and (d) SSP585 scenarios over 2036-2065. Circle sizes in each sub-figure indicate each region's planning installed capacity of wind turbine and solar PV in China’s 14th Five-Year Plan [7].


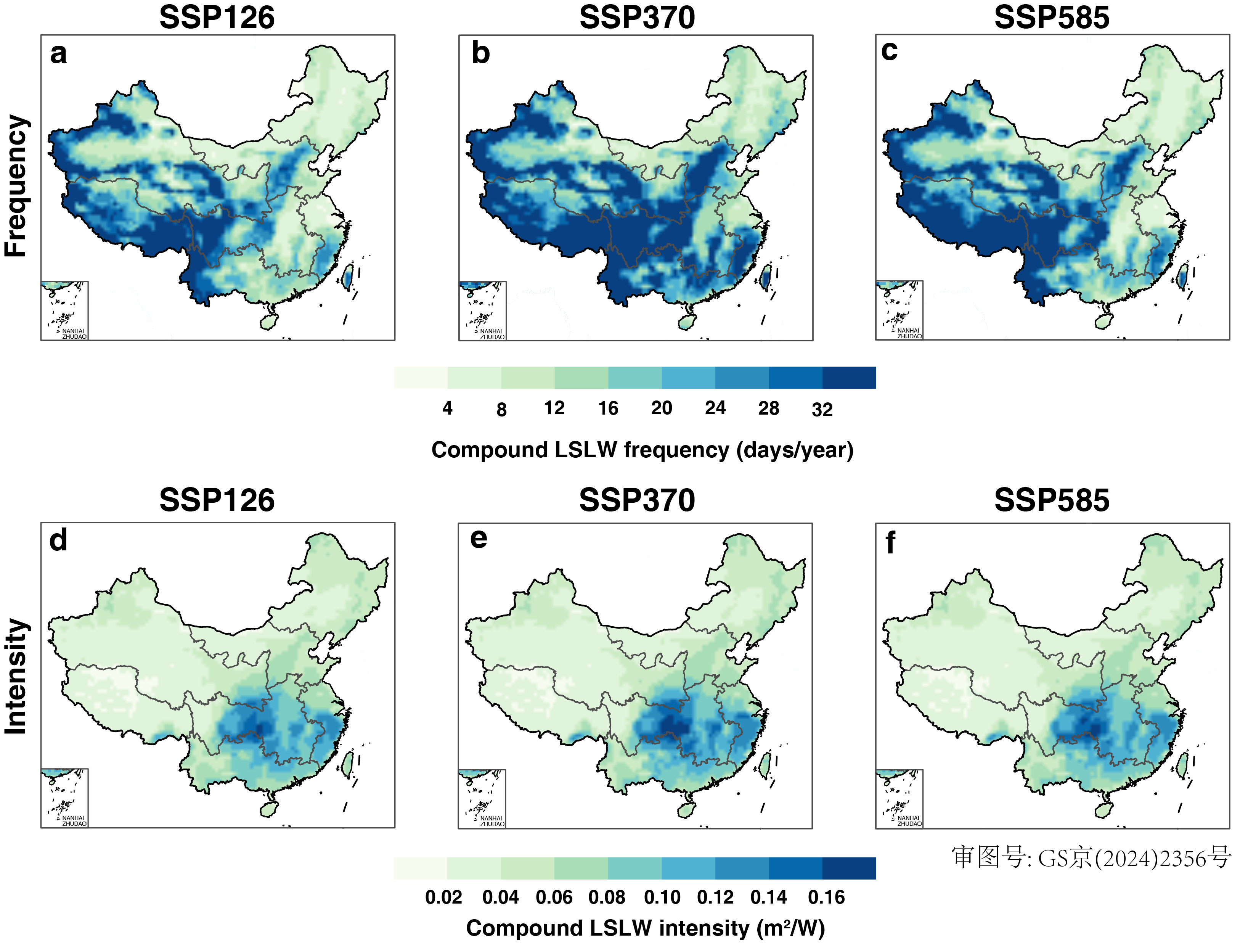


**Supplementary Figure S26.** The spatial distribution of compound low-solar-low-wind (LSLW) extremes’ (a-c) frequency (days/year) and (d-f) intensity (m^2^/W) under (a, d) SSP126, (b, e) SSP370, and (c, f) SSP585 scenarios over 2036-2065 based on multi-model ensemble mean.





**Supplementary Figure S27**. Province compound low-solar-low-wind extremes’ frequency and intensity mitigation potential. The mitigation potential for the (a) frequency and (b) intensity of compound low-solar-low-wind extremes via inter-province electricity transmission under SSP126 scenarios over 2036-2065. Gray bar shows the average mitigation potential (AMP) of inter-province electricity transmission for each province in connecting with all other provinces.


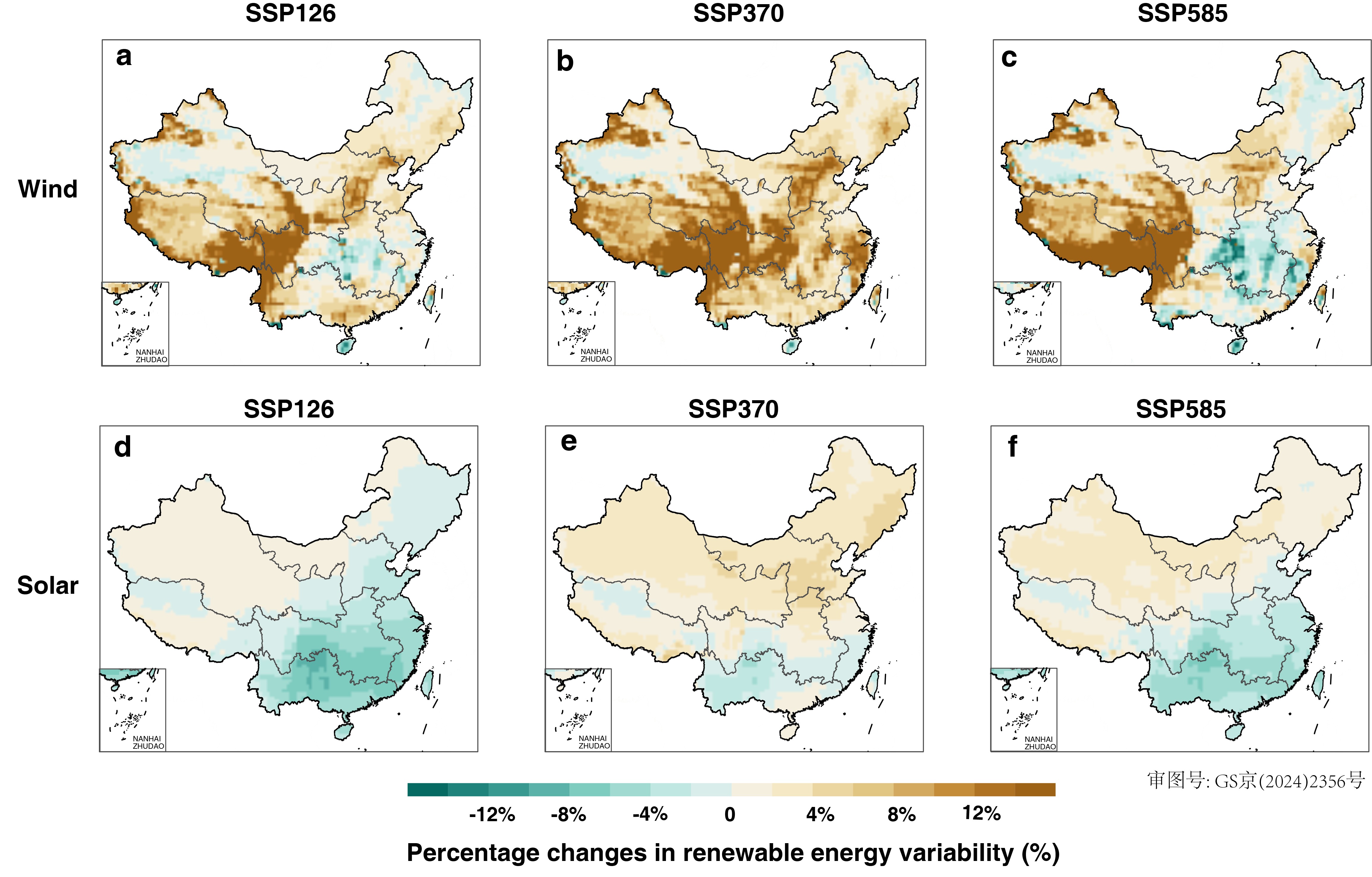


**Supplementary Figure S28**. Percentage changes (%) in the variability of (a-c) wind and (d-f) solar resource under (a, d) SSP126, (b, e) SSP370, and (c, f) SSP585 scenarios over 2036-2065 relative to the historical period (1961-1990).


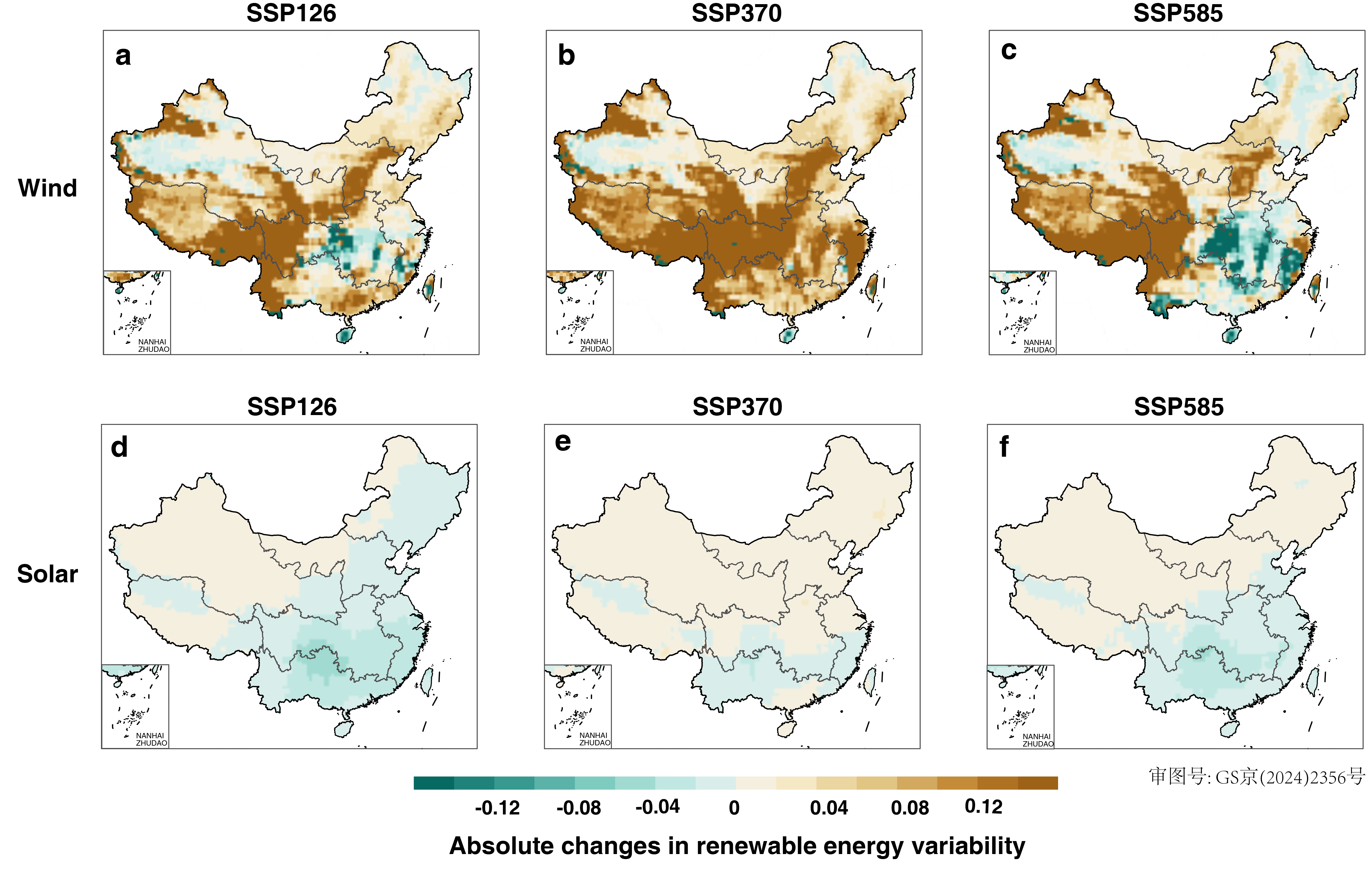


**Supplementary Figure S29**. Absolute changes in the variability of (a-c) wind and (d-f) solar resource under (a, d) SSP126, (b, e) SSP370, and (c, f) SSP585 scenarios over 2036-2065 relative to the historical period (1961-1990).


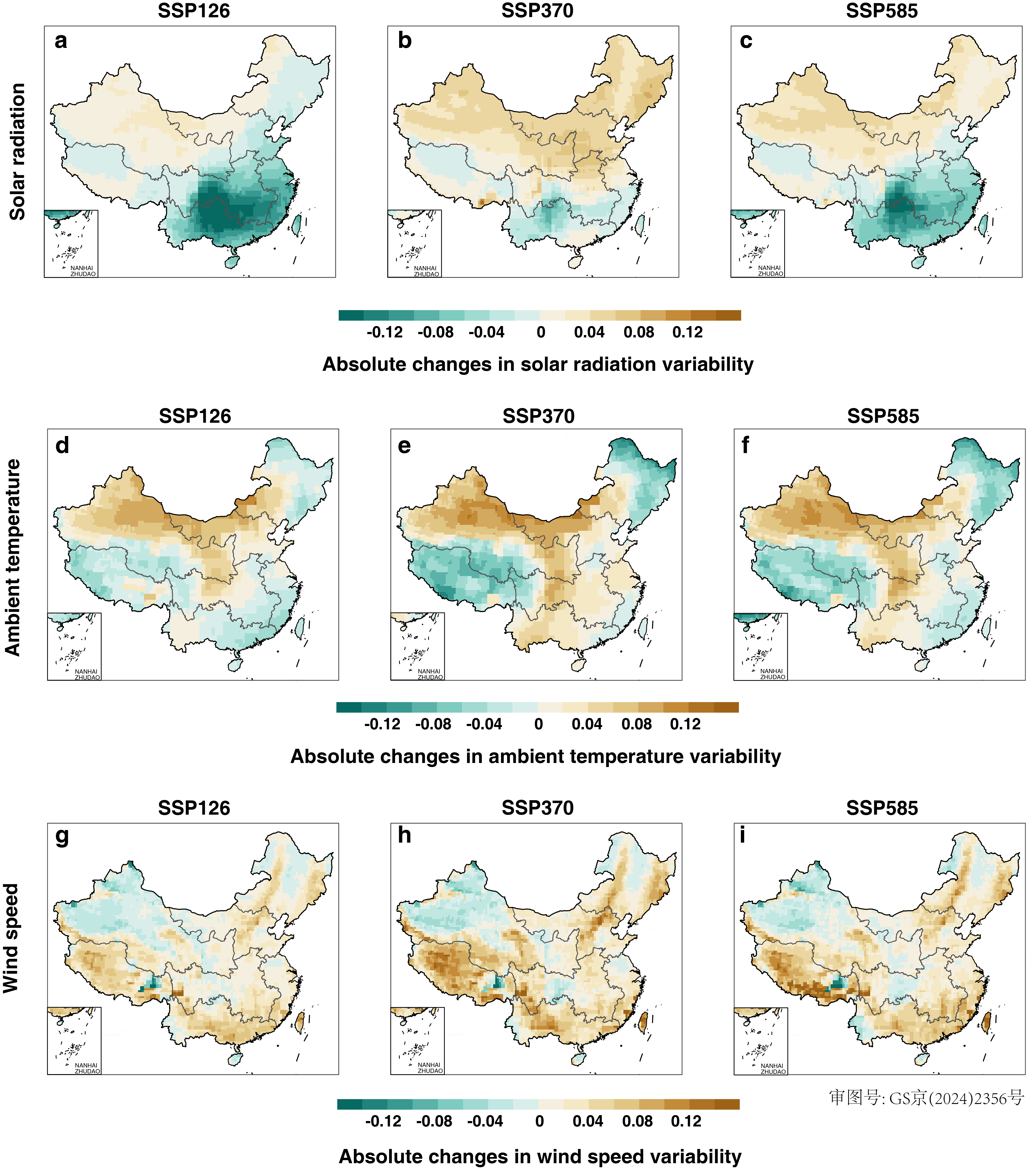


**Supplementary Figure S30**. Absolute changes in the variability of (a-c) solar radiation, (d-f) temperature, and (g-i) wind speed under (a, d, g) SSP126, (b, e, h) SSP370, and (c, f, i) SSP585 scenarios over 2036-2065 relative to the historical period (1961-1990).


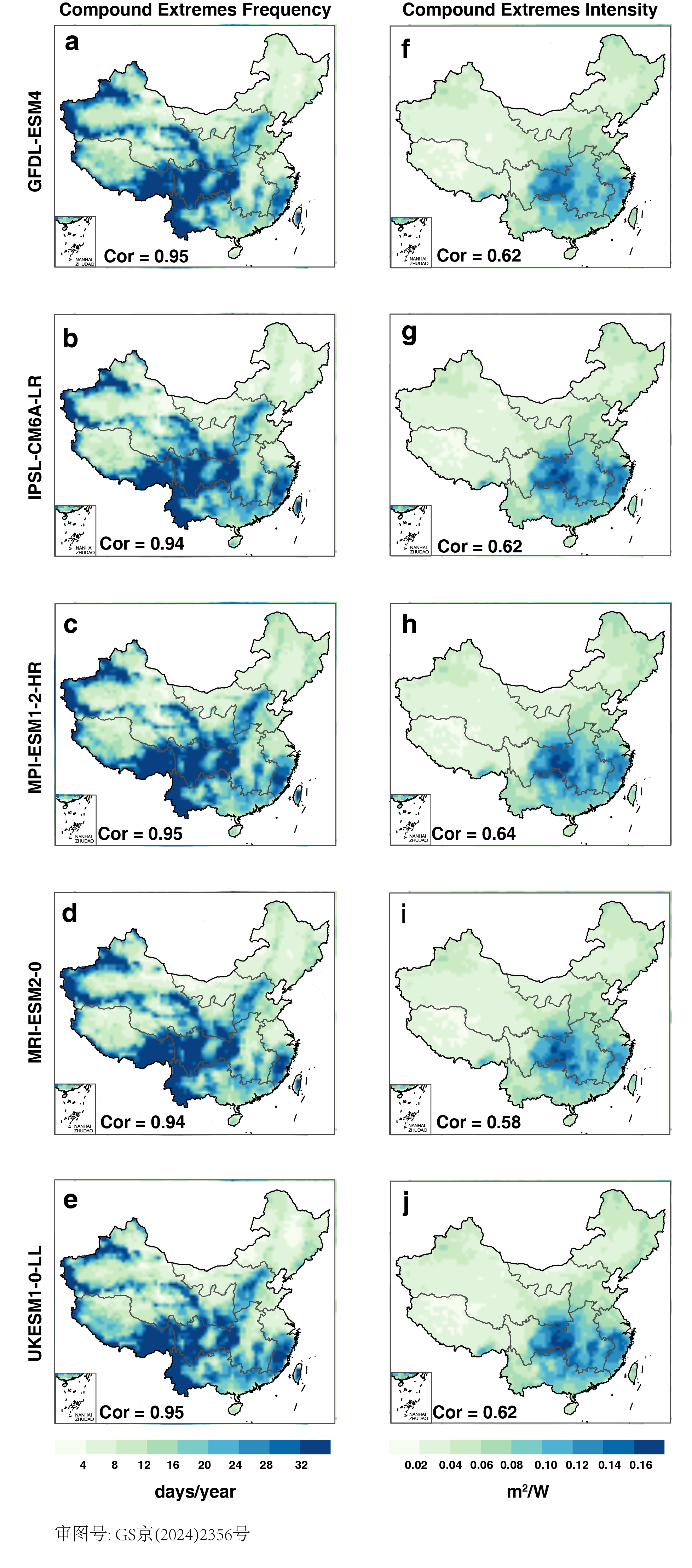


**Supplementary Figure S31**. The spatial distribution of compound low-solar-low-wind extremes’ (a-e) frequency (days/year) and (f-j) intensity (m^2^/W) for individual CMIP6 model over the historical period (1961-1990). The correlation value represents the spatial correlation of compound low-solar-low-wind extremes’ frequency or intensity between the individual CMIP6 model and the ERA5 reanalysis dataset.


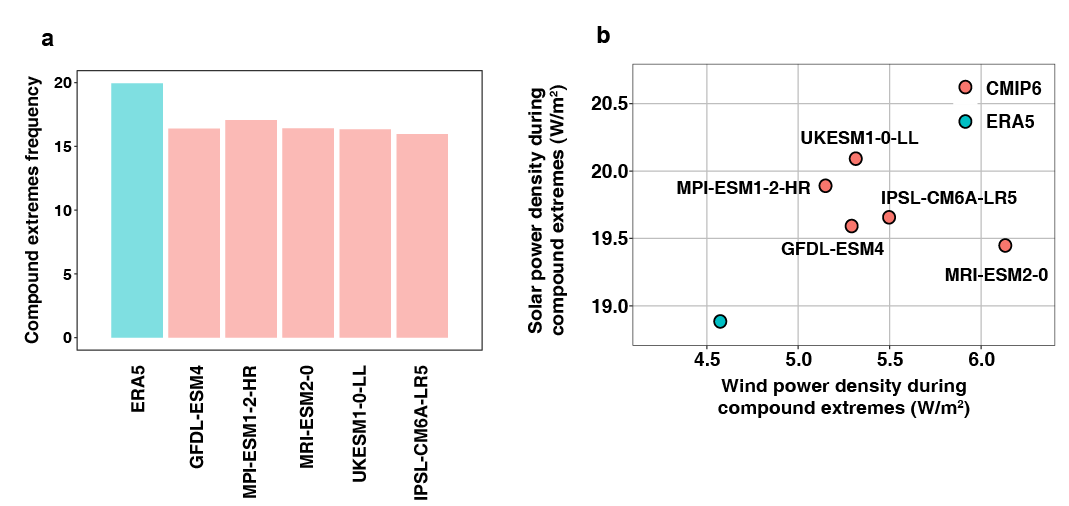


**Supplementary Figure S32**. (a) Compound low-solar-low-wind extremes’ frequency (days/year) averaged across the country during the period of 1961-1990 (individual CMIP6 models, red histogram) and 1981-2010 (ERA5, blue histogram). (b) Wind and solar power density across the country during compound low-solar-low-wind extremes over the period 1961-1990 (individual CMIP6 models, red points) and 1981-2010 (ERA5, blue point).


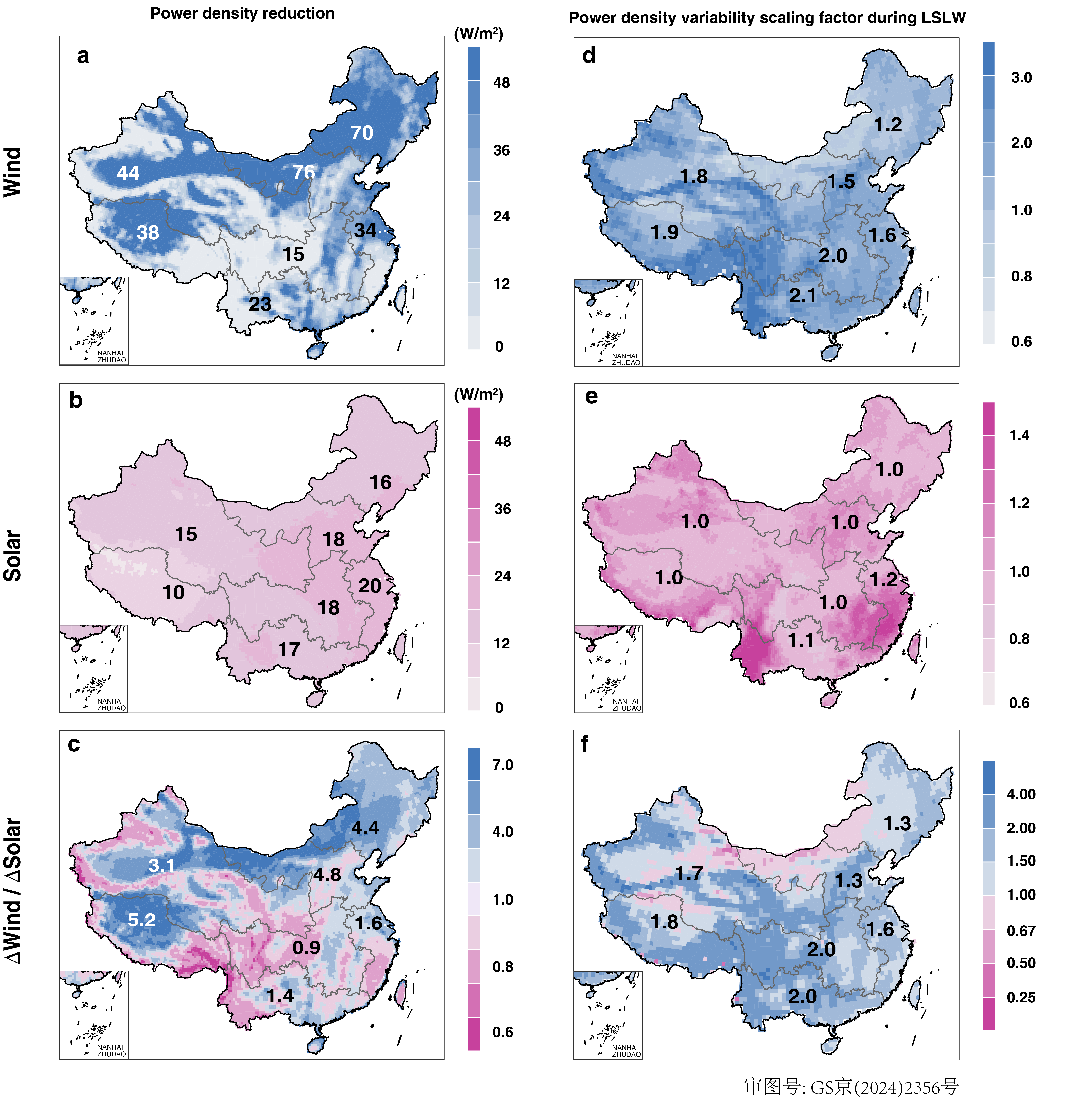


**Supplementary Figure S33.** Wind and solar power changes during historical compound low-solar-low-wind (LSLW) extremes (1961-1990) based on ERA5 reanalysis dataset. The spatial distribution of (a) wind and (b) solar resource reduction (power density under average climate minus that under LSLW, RE_average climate_-RE_LSLW_), and the scaling factor of (d) wind and (e) solar resource variability (power density variability under LSLW divided by that under average climate, CV_LSLW_/CV_average climate_), as well as the relative importance between wind and solar in (c) resource reduction ((WE_average climate_-WE_LSLW_)/(PV_average climate_-PV_LSLW_)) and (f) variability changes ((CV_WE LSLW_/CV_WE average climate_)/( CV_PV LSLW_/CV_PV average climate_)) during compound LSLW extremes relative to average climate.


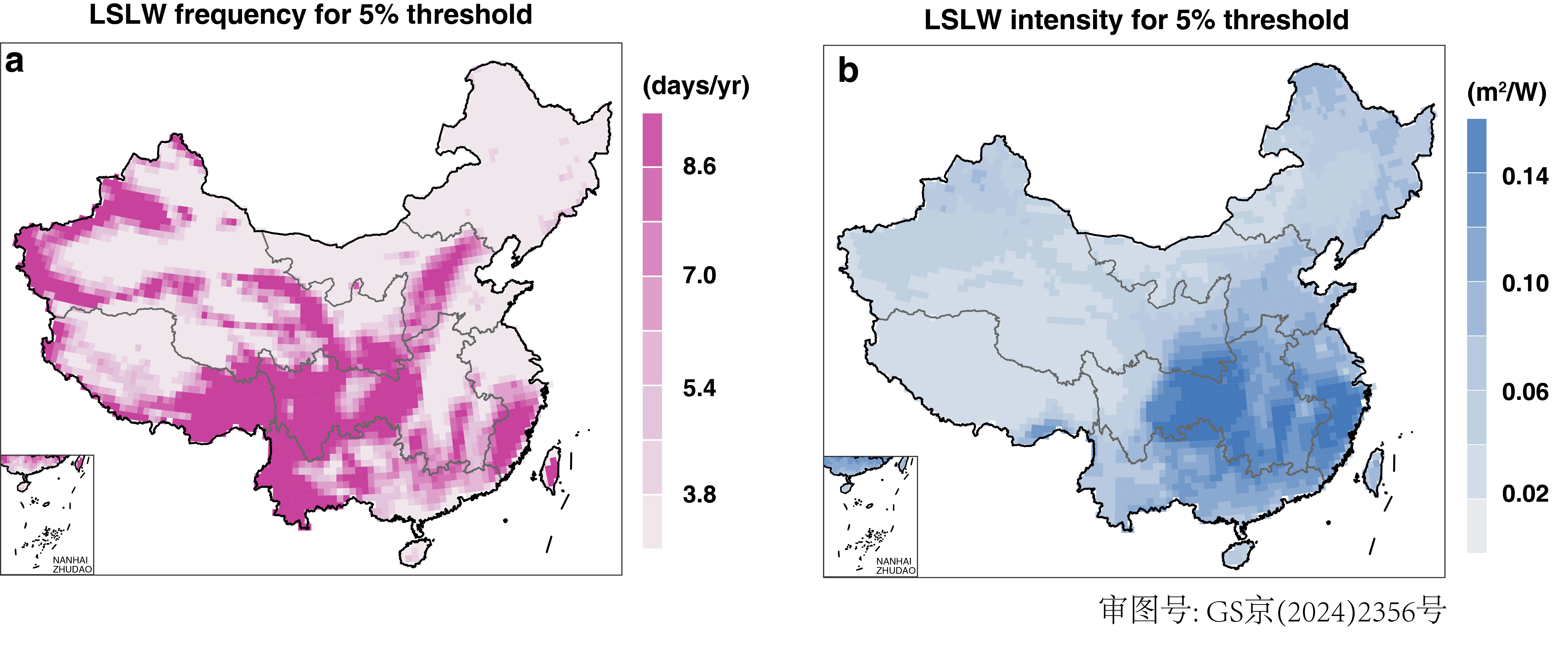


**Supplementary Figure S34.** The spatial distribution of compound low-solar-low-wind (LSLW) extremes’ (a) frequency (days/yr) and (b) intensity (m^2^/W) for the 5^th^ percentile threshold during the historical period (1961-1990).


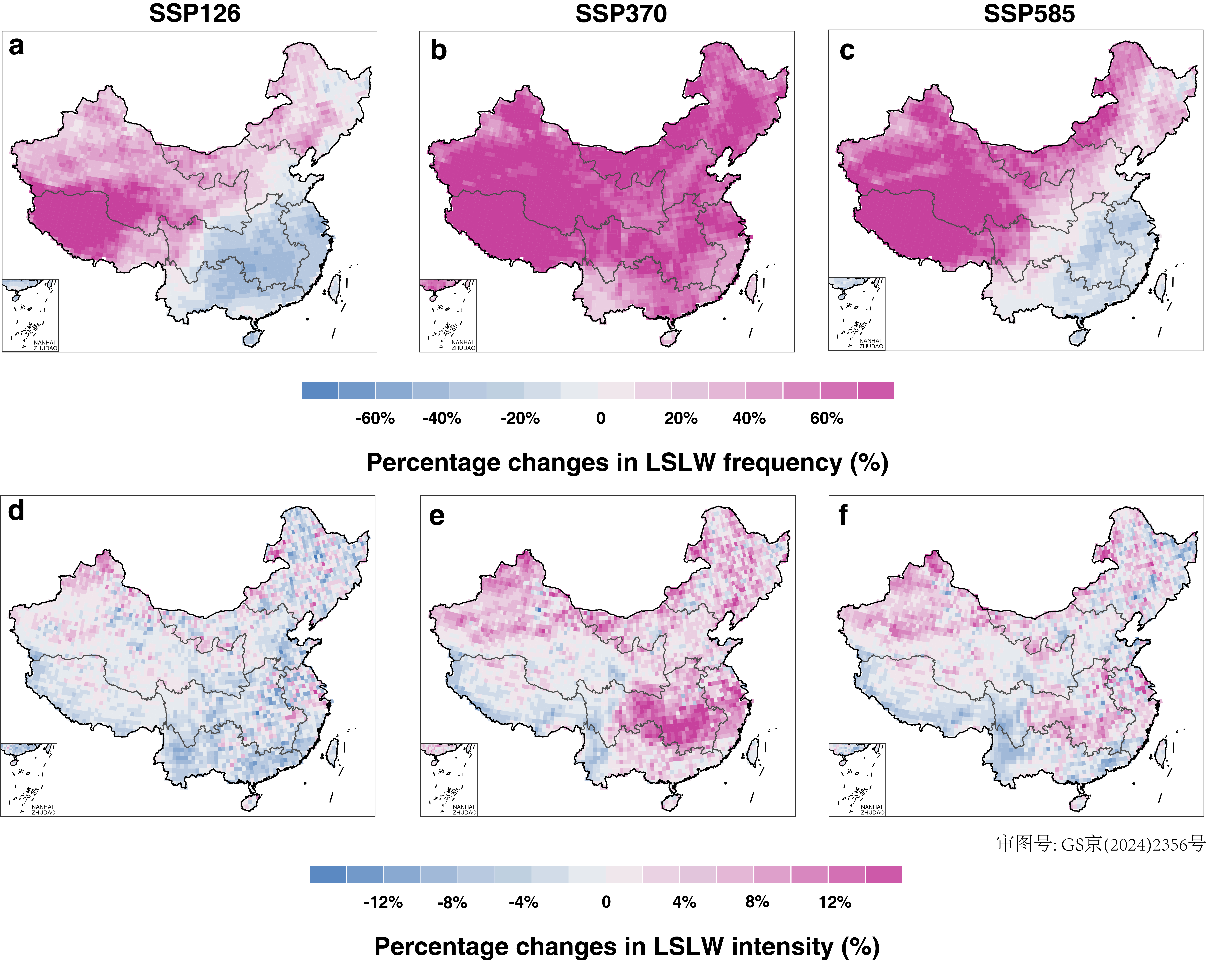


**Supplementary Figure S35**. Percentage changes (%) in the compound low-solar-low-wind (LSLW) extremes’ (a-c) frequency and (d-f) intensity for the 5th percentile threshold under (a, d) SSP126, (b, e) SSP370, and (c, f) SSP585 scenarios over 2036-2065 relative to the historical period (1961-1990).


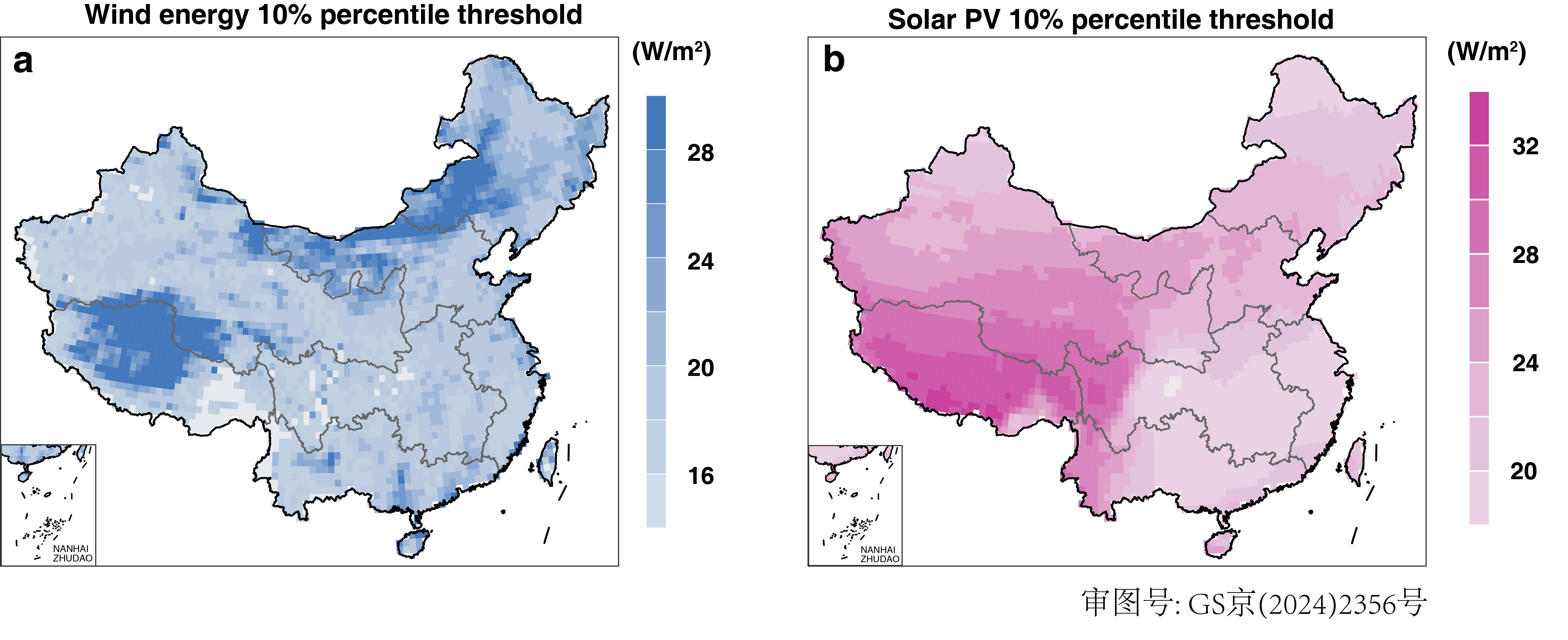


**Supplementary Figure S36**. The spatial distribution of 10^th^ percentile threshold of (a) wind energy (a, W/m^2^) and (b) solar PV (W/m^2^) during the historical period (1961-1990).


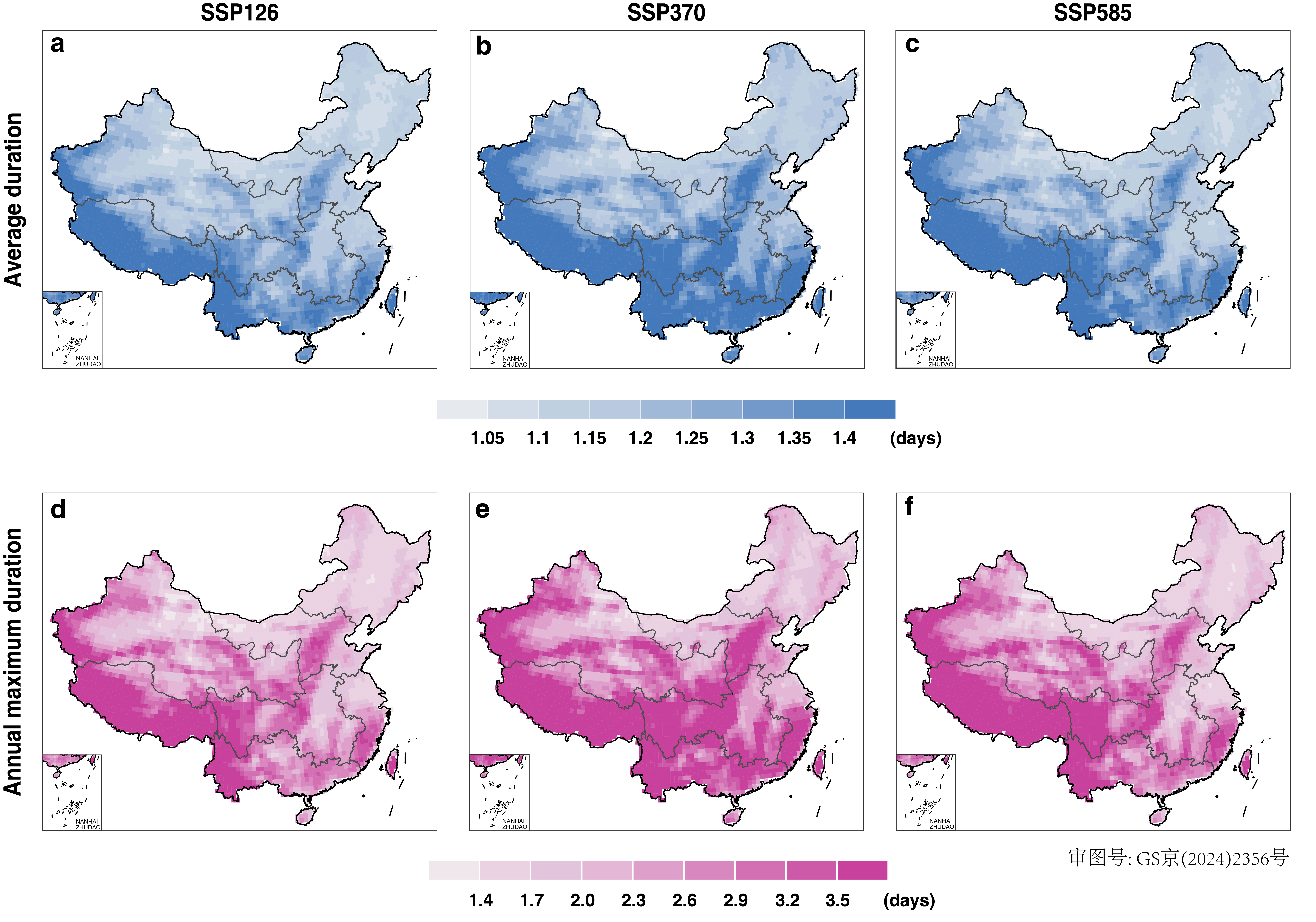


**Supplementary Figure S37.** The spatial distribution of compound low-solar-low-wind (LSLW) extremes’ (a) average duration (days) and (b) annual maximum duration (days) under (a, d) SSP126, (b, e) SSP370, and (c, f) SSP585 scenarios over 2036-2065.

Supplementary Table 1 Regional average compound LSLW extremes’ frequency (days/yr)

| Power grid | Historical | SSP126 | SSP370 | SSP585 |
| --- | --- | --- | --- | --- |
| Northeast | 7.41 | 8.11 | 11.03 | 9.06 |
| Northwest | 17.10 | 21.44 | 27.46 | 25.69 |
| North | 12.02 | 13.23 | 18.12 | 14.53 |
| Xizang | 19.66 | 31.66 | 40.73 | 43.56 |
| Central | 23.30 | 21.09 | 32.64 | 24.85 |
| East | 15.48 | 12.39 | 19.91 | 13.57 |
| South | 23.02 | 19.82 | 29.26 | 22.39 |

**Reference**

1. Lei Y, Wang Z, Wang D *et al.* Co-benefits of carbon neutrality in enhancing and stabilizing solar and wind energy. *Nat Clim Chang* 2023:693–700.

2. Tong D, Farnham DJ, Duan L *et al.* Geophysical constraints on the reliability of solar and wind power worldwide. *Nat Commun* 2021;**12**:6146.

3. Liu L. Climate change impacts on planned supply–demand match in global wind and solar energy systems. *Nature Energy*.

4. Feron S, Cordero RR, Damiani A *et al.* Climate change extremes and photovoltaic power output. *Nat Sustain* 2021;**4**:270–6.

5. Jerez S, Tobin I, Vautard R *et al.* The impact of climate change on photovoltaic power generation in Europe. *Nat Commun* 2015;**6**:10014.

6. Ridder NN, Pitman AJ, Westra S *et al.* Global hotspots for the occurrence of compound events. *Nat Commun* 2020;**11**:5956.

7. National Development and Reform Commission, 2022. *Fourteenth Five-Year Plan on Renewable Energy Development.* https://www.ndrc.gov.cn /xxgk/zcfb/ghwb/202206/t20220601_1326719.html?code=&state=123 (12 June 2024, date last accessed).
